# Long‐Life Aqueous Organic Redox Flow Batteries Enabled by Amidoxime‐Functionalized Ion‐Selective Polymer Membranes

**DOI:** 10.1002/anie.202207580

**Published:** 2022-08-09

**Authors:** Chunchun Ye, Rui Tan, Anqi Wang, Jie Chen, Bibiana Comesaña Gándara, Charlotte Breakwell, Alberto Alvarez‐Fernandez, Zhiyu Fan, Jiaqi Weng, C. Grazia Bezzu, Stefan Guldin, Nigel P. Brandon, Anthony R. Kucernak, Kim E. Jelfs, Neil B. McKeown, Qilei Song

**Affiliations:** ^1^ Department of Chemical Engineering Imperial College London London SW7 2AZ UK; ^2^ EaStCHEM School of Chemistry University of Edinburgh Edinburgh EH9 3FJ UK; ^3^ Department of Chemistry Molecular Sciences Research Hub Imperial College London London W12 0BZ UK; ^4^ Department of Chemical Engineering University College London London WC1E 7JE UK; ^5^ Department of Earth Science and Engineering Imperial College London London SW7 2AZ UK

**Keywords:** Energy Storage, Ion-Exchange Membranes, Microporous Polymers, Redox Flow Batteries, Separation Membranes

## Abstract

Redox flow batteries (RFBs) based on aqueous organic electrolytes are a promising technology for safe and cost‐effective large‐scale electrical energy storage. Membrane separators are a key component in RFBs, allowing fast conduction of charge‐carrier ions but minimizing the cross‐over of redox‐active species. Here, we report the molecular engineering of amidoxime‐functionalized Polymers of Intrinsic Microporosity (AO‐PIMs) by tuning their polymer chain topology and pore architecture to optimize membrane ion transport functions. AO‐PIM membranes are integrated with three emerging aqueous organic flow battery chemistries, and the synergetic integration of ion‐selective membranes with molecular engineered organic molecules in neutral‐pH electrolytes leads to significantly enhanced cycling stability.

## Graphical abstract

Highly ion‐selective robust membranes are developed from a series of amidoxime‐functionalized Polymers of Intrinsic Microporosity (AO‐PIMs), which enable long lifetime operation of aqueous redox flow batteries (RFBs) using low‐cost organic redox molecules at benign near‐neutral pH, offering cost‐effective electricity storage for grid‐scale renewable power sources.

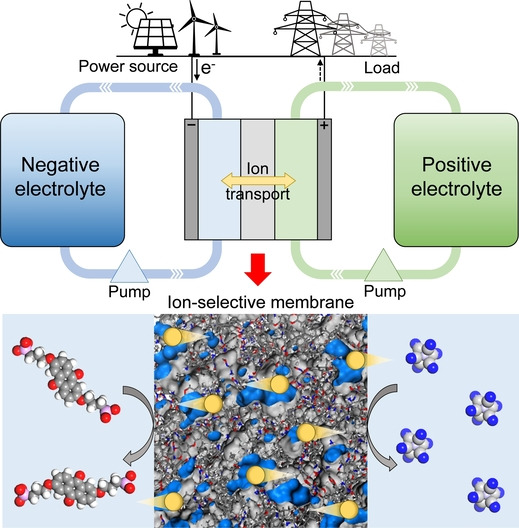

## Introduction

The increasing demand for sustainable and renewable energy resources, e.g., solar and wind power, requires the development of efficient electrical energy storage (EES) technologies.^[1]^ Redox flow batteries (RFBs) are a promising EES technology for safe and cost‐effective energy storage.^[2]^ RFBs typically consist of two compartments, where active materials undergo reversible reactions, as well as a membrane that provides an electric‐insulating, ion‐selective barrier separating the two compartments. Membrane ion transport properties are the critical parameters that determine the efficacy of RFB systems by facilitating fast transport of charge‐carrier ions while avoiding crossover‐mixing of redox‐active species.^[3]^ Highly conductive and selective membranes are particularly desirable for emerging RFB chemistries that utilize small molecular redox‐active species.^[4]^

Commercial perfluorosulfonic acid (PFSA) membranes, such as Nafion, are expensive and involve the use of polyfluoroalkyl substances (PFASs, known as “forever chemicals”) in their production. Significant efforts have been devoted to the development of alternative membranes from low‐cost hydrocarbon polymers, such as polybenzimidazole^[5]^ and sulfonated poly(ether ether ketone) (SPEEK).^[6]^ However, it remains challenging to overcome the trade‐off between ionic conductivity and selectivity, i.e., highly permeable/conductive membranes possess low selectivity and vice versa.^[7]^ Nanofiltration membranes have also been used as membrane separators for vanadium flow batteries, but their selectivity is insufficient for aqueous organic flow batteries due to their relatively large pore size.^[8]^

Microporous materials are emerging materials for making membranes with high selectivity based on their well‐defined channels,^[9]^ such as Metal‐Organic Frameworks (MOFs)^[10]^ and Covalent‐Organic Frameworks (COFs).^[11]^ However, the poor processability of these crystalline materials makes them difficult to manufacture especially for large area membranes. In contrast, Polymers of Intrinsic Microporosity (PIMs) combine microporosity and solution‐processability,^[12]^ and can be readily processed into robust membranes that break the trade‐off between guest species permeability and selectivity, particularly for gas separation applications.^[13]^ Recently, PIM membranes have been demonstrated in electrochemical devices and show promising performance in fuel cells,^[14]^ flow batteries,^[15]^ lithium–sulfur^[16]^ and lithium‐metal batteries.^[17]^ Of particular interest are PIMs functionalized with ionizable amidoxime (AO) groups that can be deprotonated at high pH (p*K*_a_=13.2).^[18]^ AO‐PIMs have been used as membrane separators in alkaline aqueous organic RFBs,^[19]^ but the degradation of active materials and mechanical failure of polymer membranes in alkaline electrolytes limit the battery performance.^[20]^ For RFBs to mature as a deployable grid storage technology, both membrane separators and active molecules require further engineering to extend the longevity of RFBs.

**Figure 1:**
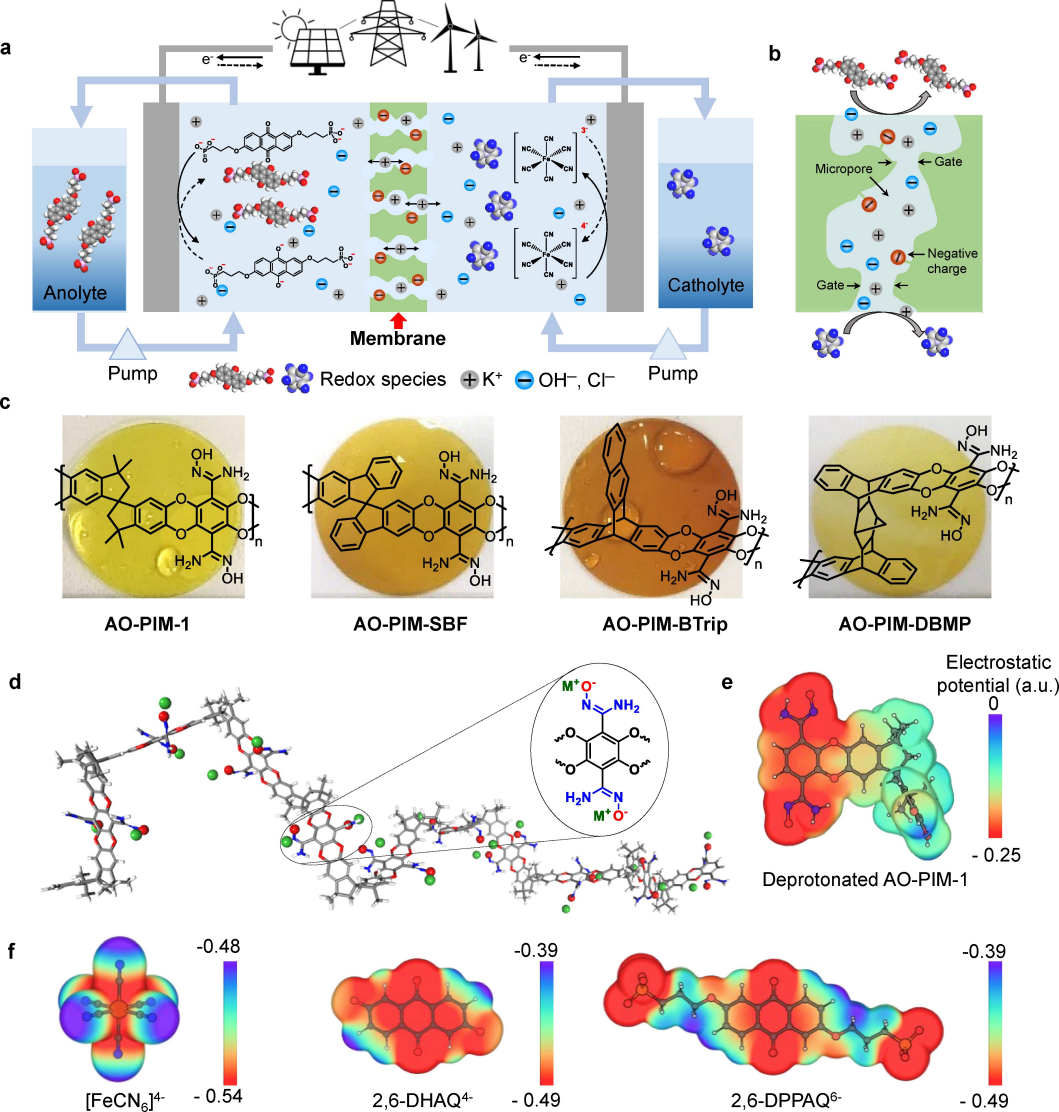
a) Schematic illustration of an aqueous organic redox flow battery for grid‐scale energy storage. K_4_Fe(CN)_6_ and 2,6‐DPPAQ are shown as model redox‐active materials operated at near‐neutral pH electrolytes. Grey and blue balls are described as charge‐carrier ions, and red balls as AO groups, respectively. b) Schematic illustration of interconnected pores with the sub‐nanometre size that enable fast transport of charge‐carrying ions (e.g., K^+^ and Cl^−^) while blocking relatively large redox‐active materials. c) Chemical structures and photographs of corresponding 50‐μm‐thick self‐standing membranes of AO‐PIMs including AO‐PIM‐1, AO‐PIM‐SBF, AO‐PIM‐BTrip and AO‐PIM‐DBMP, respectively. The membrane diameter is ≈20 mm. d) Molecular model of AO‐PIM‐1 shows the rigid and contorted PIM backbones with deprotonated AO groups. Electrostatic potential (ESP) of e) AO‐PIM‐1 repeating units and f) redox‐active molecules including [Fe(CN_6_)]^4−^, 2,6‐DHAQ^4−^, and 2,6‐DPPAQ^6−^.

Here, we report the optimization of the ion‐transport selectivity in AO‐PIMs through rational design of polymer chain topology and pore architecture, as well as their synergetic integration with redox active species in benign near neutral‐pH electrolyte for long‐cycle life, crossover‐free redox flow batteries (Figure 1a). Spirocyclic and bridged bicyclic structural units were employed to afford AO‐PIMs composed of either 2D or 3D macromolecular chains, so as to finely tune the geometry, architecture and size of the micropores that result from the inefficient packing of these polymer chains in the solid state (Figure 1b–d and Figure S1). Ionic and molecular transport functions of these AO‐PIMs were systematically investigated, establishing the fundamental correlations between polymer chain structure, pore architecture and ion transport. The size‐sieving effect is particularly effective for molecules with relatively large physical size, while the Donnan exclusion effect further enhances molecular selectivity based on the same negative charge properties between activated AO groups within PIM backbones and active materials (Figure 1e, f, Figure S2 and Table S1). AO‐PIMs are solution‐processed into mechanically robust, self‐standing membranes and show high ionic conductivity up to 30.7 mS cm^−1^ in 1 M KOH and effective blocking of negative‐charged redox‐active molecules. AO‐PIMs are well positioned to provide a long‐cycling lifetime for aqueous organic RFBs in benign near neutral‐pH electrolytes. Our membrane design strategy may inspire the development of a new generation of size‐selective ion‐exchange membranes for a wide range of electrochemical processes for energy and environmental applications.

## Results and Discussion

We prepared AO‐PIMs through an efficient dibenzodioxin‐forming polymerization reaction followed by a nitrile‐to‐amidoxime modification reaction under mild conditions. Aiming to vary the architecture and topology of micropores and water clusters in the AO‐PIMs, we employed four different structural units including spirocyclic spirobisindane (SBI) and spirobifluorene (SBF) units that afford a 3D polymer chain configuration, as well as highly rigid bridged bicyclic benzotriptycene (BTrip) and dibenzomethanopentacene (DBMP) units that give a 2D polymer chain configuration. These polymers were characterized by FTIR,^13^C NMR, and TGA measurements. The details of synthesis and characterization are given in the Supporting Information (Figures S3–S5 and Table S2). All four AO‐PIMs are soluble in polar organic solvents including DMSO, DMF, DMAc and NMP, allowing ease of fabrication via solution casting to afford mechanically robust membranes (Figure S6 and S7 and Table S3) as well as characterization of molecular weight using GPC (Figure S8).

**Figure 2:**
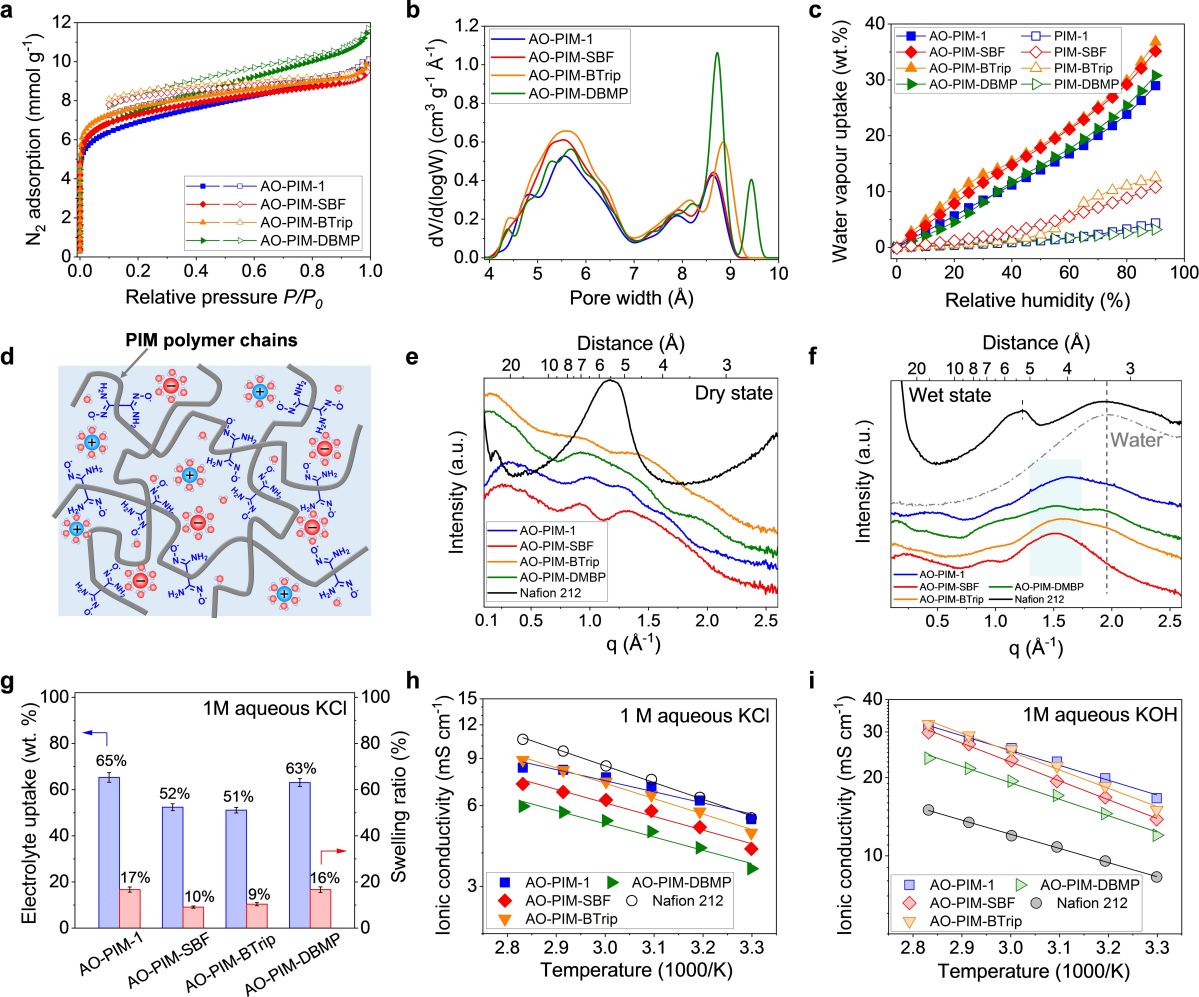
a) N_2_ adsorption–desorption isotherms of AO‐PIMs at 77 K. Solid symbols: adsorption. Open symbols: desorption. b) Pore size distribution of AO‐PIMs based on DFT calculation derived from CO_2_ adsorption‐desorption isotherms at 273 K. c) Water vapour uptake isotherms as a function of relative humidity at 25 °C of AO‐PIM (solid symbols) and corresponding parent PIM membranes (open symbols). d) Schematic diagram showing narrow ion transport channels generated by rigid and contorted PIM polymer chains armed with activated AO groups. e) WAXS patterns of AO‐PIM and Nafion 212 membranes in dry states and f) wet states. g) Electrolyte uptake and linear swelling ratio in 1 M aqueous KCl of AO‐PIM membranes. Error bars are standard deviations derived from three measurements based on three different samples. Temperature dependence of ionic conductivity in the range of 30–80 °C for AO‐PIM and Nafion 212 membranes measured in h) 1 M aqueous KCl and i) 1 M aqueous KOH using deprotonated AO‐PIM membranes.

N_2_ sorption isotherms of these PIMs exhibited high capacity at low relative pressure and apparent Brunauer–Emmett–Teller surface area (SA_BET_) calculated from these isotherms were in the range of 550–650 m^2^ g^−1^ (Figure 2a, Figure S9a–d and Table S4). Adsorption of CO_2_ at 273 K displayed enhanced capacities for AO‐PIMs relative to the pristine PIMs (Figure S9e–h), due to the variation in microstructure and stronger Lewis acid‐base interactions between CO_2_ and AO groups.^[21]^ The pore size of all four AO‐PIMs exhibited a narrow distribution in the range of 4 to 7 Å with the most frequent pore width being 5.6 Å (Figure 2b). Compared with pristine PIMs, AO functionalization led to a shift in the pore size towards the ultra‐microporous range (<7Å) and a reduction in larger micropores (>7 Å) (Figure S9i–l). Importantly, polymer chain topology was found to have a critical effect on the pore size of these AO‐PIMs. Compared with AO‐PIM‐1, AO‐PIM‐SBF with a more rigid chain structure showed a greater portion of ultra‐micropores, which may be beneficial for improving the size selectivity towards ions. In contrast, AO‐PIM‐DBMP showed a significant portion of larger sub‐nm pores owing to the bulky structure of the DBMP structural unit, while AO‐PIM‐BTrip exhibited a greater portion of both smaller pores and larger pores. The variation in pore size distribution and polymer chain topology will have a significant influence on the geometry of water clusters formed within the free volume elements as well as ion transport properties. Charge‐neutral AO‐PIM membranes showed a water vapour sorption capacity in the range of 21–27 wt.%, while deprotonated AO‐PIM polymers with higher hydrophilicity exhibited higher capacity in the range of 29–37 wt.%. In contrast, despite high surface areas and high total pore volumes, all pristine PIM membranes showed very low uptake of water vapour due to their hydrophobicity and poor wettability (Figure 2c, Figure S10 and Table S4).

The pore structure in both dry and water‐swollen states was further investigated using small‐angle X‐ray scattering (SAXS, Figure S11) and wide‐angle X‐ray scattering (WAXS, Figure S12). In SAXS patterns, a broad ionomer peak at q≈0.15 Å^−1^ (≈4.2 nm in real space) was observed for wet Nafion 212 membrane, corresponding to the spacing of hydrophilic water domains.^[22]^ In contrast, there was no significant scattering feature observed for AO‐PIM membranes over a wide low‐q range from 0.05 to 0.3 Å^−1^ in both dry and wet states, indicating the absence of mesoscale phase separation. Further, broad scattering peaks were observed at q≈0.9–2.0 Å^−1^ (7.2–3.2 Å in real space) in WAXS for dry AO‐PIM membranes (Figure 2e), which may result from their sub‐nanometer channels. When fully hydrated, AO‐PIM membranes showed broad peaks with q position centred on ≈1.5 Å^−1^ (Figure 2f), presumably arising from the sub‐nanometre water domains formed within their intrinsic micropores, while the sharp scattering peak at q≈1.17 Å^−1^ for Nafion corresponded to the crystallinity of its polymer backbone.

The ion dynamics and conductivity in polymer electrolyte membranes depend heavily on the adsorption of salt electrolytes and the interaction of salt ions with polymers (Figure 2d). Generally, AO‐PIM membranes show significant water/electrolytes uptake (51–65 wt.%) with a relatively small degree of swelling (10–17 %) (Figure 2g, Figure S13 and Table S5). The low swelling ratio indicates that the sub‐nanometer pores are retained in the membranes and provide the nanoscale confinement effect on ion transport. The ionic conductivity of 50‐μm‐thick AO‐PIM membranes was measured by electrochemical impedance spectroscopy (EIS) in the temperature range of 30–80 °C (Figure S14). Fully deprotonated AO‐PIM membranes showed high ionic conductivity in both neutral and alkaline aqueous electrolytes, with values comparable to those of Nafion 212 membranes in 1 M aqueous KCl (Figure 2h) but higher in 1 M aqueous KOH (Figure 2i). For example, the AO‐PIM‐1 membrane showed ionic conductivity values of 16.7 mS cm^−1^ at 30 °C and 30.7 mS cm^−1^ at 80 °C in 1 M KOH solution, much higher than the values for Nafion 212 (8.3 and 15.0 mS cm^−1^ at 30 and 80 °C, respectively). A comparison of ionic conductivity of non‐deprotonated (inactivated) AO‐PIMs in 1 M KCl and deprotonated (activated) AO‐PIMs in deionized water suggests that the high ionic conductivity of fully deprotonated AO‐PIM membranes can be attributed to the combined microporosity resulting from the PIM structures, hydrophilicity from the AO functionality, and interaction between charge carriers and ionizable AO groups (Figure S15 and Table S6).

**Figure 3:**
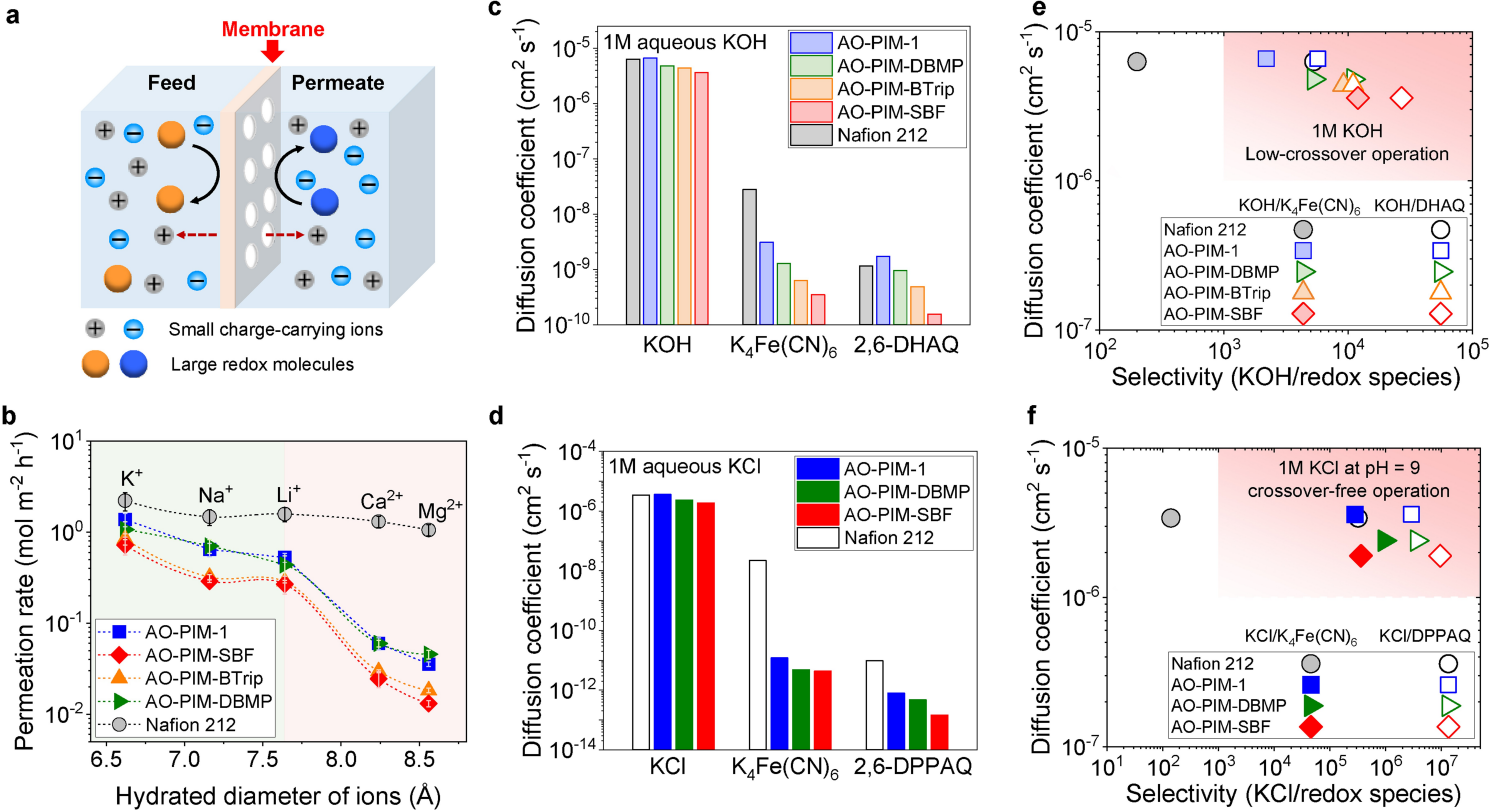
a) Diagram of selective ion transport through membranes. b) Ion permeation rates through non‐deprotonated AO‐PIM membranes with thicknesses ≈50 μm measured by dialysis diffusion H‐cells (dash lines as guides to the eye). Error bars are standard deviations derived from three measurements based on three different membrane samples. Diffusion tests were performed with measured by dialysis diffusion H‐cells, using c) 1 M KOH in deionised water, 0.1 M K_4_Fe(CN)_6_ and 2,6‐DHAQ in 1 M aqueous KOH and d) 1 M KCl in deionised water, K_4_Fe(CN)_6_ and 2,6‐DPPAQ in 1 M aqueous KCl through AO‐PIM and Nafion 212 membranes. e) KOH diffusivity versus KOH/redox molecule selectivity through AO‐PIM and Nafion 212 membranes. Solid symbols: KOH/ K_4_Fe(CN)_6_ selectivity; open symbols: KOH/2,6‐DHAQ selectivity. f) KCl diffusivity versus KCl/redox molecule selectivity through AO‐PIM and Nafion 212 membranes. Solid symbols: KCl/ K_4_Fe(CN)_6_ selectivity; open symbols: KCl/2,6‐DPPAQ selectivity.

The fast and selective salt ion transport in AO‐PIM membranes is demonstrated by concentration‐driven dialysis diffusion of a variety of common salts across 50‐μm‐thick AO‐PIM membranes in non‐deprotonation, neutral‐charge form (i.e., −OH) (Figure 3a and Figure S16). All AO‐PIM membranes showed a size‐exclusion cut‐off of around 7.6 Å with the cation permeation rates being divided into two groups: small monovalent alkali metal cations (K^+^, Na^+^, Li^+^) with permeation rates in the range of 10^0^ and 10^−1^ mol m^−2^ h^−1^ and larger divalent alkaline earth metal cations (Ca^2+^, Mg^2+^) with slower permeation rates around 10^−2^ mol m^−2^ h^−1^(Figure 3b). The ideal selectivities of K^+^ over Mg^2+^ calculated from these permeation rates were in the range of 23–55, demonstrating efficient size‐sieving performance of AO‐PIM membranes. Despite the subtle size difference between Li^+^ and Mg^2+^ ions (7.60 and 8.56 Å), the AO‐PIM‐SBF membrane still achieved a reasonably high selectivity of 22. In contrast, the size sieving phenomenon was not observed for Nafion 212 membrane with negligible selectivity between different cations (Tables S7 and S8). Such superior ion sieving properties of AO‐PIM membranes are mainly attributed to the size sieving based on the precise control over their interconnected ion channels at the sub‐nanometre scale.

We further measured the molecular sieving properties of AO‐PIM membranes using redox‐active molecules including K_4_Fe(CN)_6_ (hydration diameter 0.95 nm) 2,6‐DHAQ (0.68×1.07 nm) and 2,6‐DPPAQ (0.54×2.30 nm) (Figures S17–S20, Tables S9–S11). AO‐PIM membranes allowed free transport of small ions in both alkaline and near‐neutral aqueous solution and show high KOH and KCl permeabilities comparable to those of Nafion 212 with all values in the order of 10^−6^ cm^2^ s^−1^. The diffusion of larger negatively‐charged K_4_Fe(CN)_6_ and 2,6‐DHAQ molecules in 1 M KOH solution through AO‐PIM membranes was slower with diffusion coefficients in the range 10^−9^ to 10^−10^ cm^2^ s^−1^, at least one order of magnitude lower than those of Nafion 212 membranes. The slow permeation rates of large redox‐active molecules through AO‐PIM membranes led to a remarkably high selectivity for KOH over K_4_Fe(CN)_6_ (2.2×10^3^ to 1.2×10^4^) and KOH over 2,6‐DHAQ (5.7×10^3^ to 2.7×10^4^) (Figure 3c, e). The high selectivity correlates well with the pore size distribution of AO‐PIMs measured from gas sorption. Despite a large portion of large micropores (>7Å), AO‐PIM‐DBMP showed higher ionic/molecular selectivity than AO‐PIM‐1, likely due to its greater rigidity reducing thermal motion of chain segments that form transient large voids. When applied in near‐neutral aqueous KCl (pH=9), AO‐PIM membranes exhibited even stronger blocking ability towards K_4_Fe(CN)_6_ and 2,6‐DPPAQ molecules with diffusivity in the range of 10^−11^–10^−13^ cm^2^ s^−1^ (Figure 3d, f).

**Figure 4:**
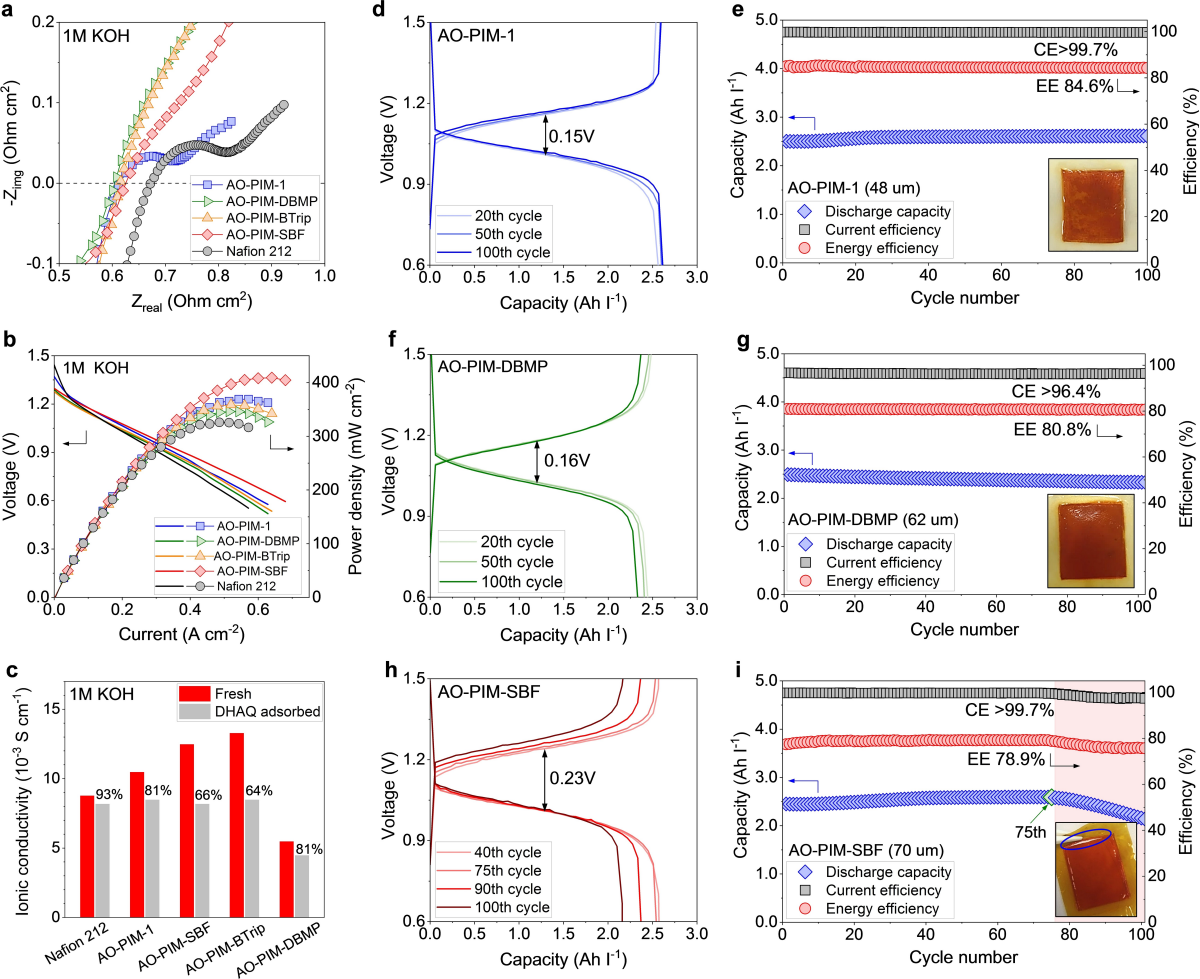
a) EIS spectra and b) polarization curves at ≈100 % SOC and 80 mA cm^−2^ measured in 0.1 M 2,6‐DHAQ|K_4_Fe(CN)_6_ RFB cells configured with AO‐PIM and Nafion 212 membranes. c) Apparent ionic conductivity values measured in 1 M aqueous KOH at 30° of fresh and DHAQ adsorbed AO‐PIM and Nafion 115 membranes. Cycling performance and representative charging‐discharging curves measured in 0.1 M 2,6‐DHAQ|K_4_Fe(CN)_6_ RFB configured with d, e) AO‐PIM‐11membrane, f, g) AO‐PIM‐DBMP membrane and h, i) AO‐PIM‐SBF membrane, respectively. Inset photographs showing the cycled AO‐PIM membranes disassembled from RFB full‐cells.

To evaluate their battery performance, 50‐μm‐thick self‐supported AO‐PIM membranes were paired with the well‐established 2,6‐DHAQ|K_4_Fe(CN)_6_ redox couple in 1 M aqueous KOH (Figure S21). AO‐PIM membranes showed lower resistance in flow cells with values between 0.604 and 0.624 Ω cm^2^ relative to that of Nafion 212 (0.672 Ω cm^2^, Figure 4a). As a result, the power density in RFBs assembled with low‐resistance AO‐PIM membranes showed higher values (370 mW cm^−2^ at 100 % SOC for AO‐PIM‐1) than that of an otherwise identical Nafion 212 cell (326 mW cm^−2^, Figure 4b and Figure S19). Energy efficiency was also high for RFBs assembled with AO‐PIM membranes (EE>80 %). For example, a battery configured with an AO‐PIM‐1 membrane afforded a coulombic efficiency (CE) of 99.7 % and a EE of 84.6 % at a current density of 60 mA cm^−2^. The AO‐PIM‐1 cell also accessed ≈96 % of the theoretical discharge capacity and showed only a small voltage gap (0.15 V, Figure 4d, e). The AO‐PIM‐DBMP RFB cell exhibited a high EE but a slightly lower CE (96.4 %) and a capacity decay from 2.44 Ah l^−1^ at the 20^th^ cycle to 2.33 Ah l^−1^ at the 100^th^ cycle (Figure 4f, g). However, both AO‐PIM‐SBF and AO‐PIM‐BTrip cells showed fast capacity fading after a few cycles of operation due to mechanical failure of the membranes (Figure 4h, i and Figure S23). AO‐PIM‐1 and AO‐PIM‐DBMP membranes disassembled from the 100‐cycle cells appeared intact, while obvious cracks could be found for AO‐PIM‐SBF and AO‐PIM‐BTrip at the edge of the active area. These cracks are likely due to the stress induced by the higher hydration at the active area and the de‐swelling and shrinkage of the boundary areas that are sandwiched between gaskets.^[23]^ No sign of polymer chemical change or degradation was found by FTIR and GPC analysis (Figure S24 and Table S12); the conductivity of AO‐PIM membranes also remained unchanged during an ageing test of over 180 days (Figure S25 and Table S13). In addition, we also observed severe fouling of AO‐PIM membranes after flow battery tests, suggesting the excessive adsorption of 2,6‐DHAQ (Figures 4e, g, i, and Figure S23a). Ionic conductivity for AO‐PIM membranes that were soaked in 2,6‐DHAQ solutions dropped to around 20 % for AO‐PIM‐1 and AO‐PIM‐DBMP and around 40 % for AO‐PIM‐SBF and AO‐PIM‐BTrip (Figure 4c, Figure S26 and Table S14), which may be due to the adsorbed 2,6‐DHAQ occupying the micropores and blocking ion transport pathways. The small molecular size of 2,6‐DHAQ as well as potential cohesive interactions (e.g., π‐π interactions) between the aromatic 2,6‐DHAQ and the polymer membranes may be the dominating driving force of the undesired adsorption.

**Figure 5:**
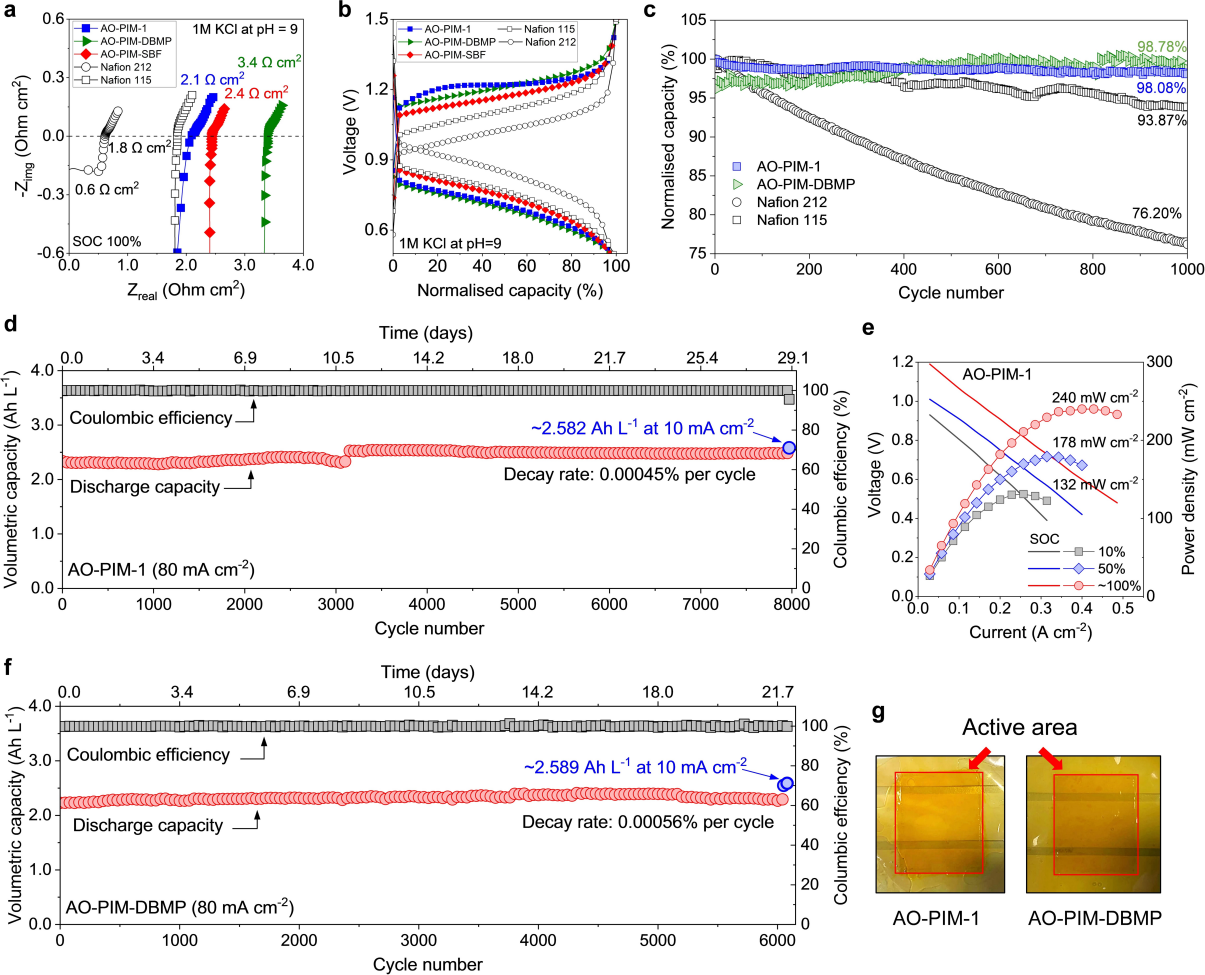
a) EIS spectra and b) polarization curves at ≈100 % SOC and 80 mA cm^−2^ measured in 0.1 M 2,6‐DPPAQ|K_4_Fe(CN)_6_ RFB cells in near‐neutral condition (pH=9) using AO‐PIM, Nafion 212 and Nafion 115 membranes. c) The comparison of cycling performance with the same cycle number of 1000 using AO‐PIM, Nafion 212 and Nafion 115 membranes in 0.1 M 2,6‐DPPAQ|K_4_Fe(CN)_6_ RFB cells at pH=9 and a current density of 80 mA cm^−2^. Long‐term cycling stability using d) AO‐PIM‐1 and f) AO‐PIM‐DBMP membranes in 0.1 M 2,6‐DPPAQ|K_4_Fe(CN)_6_ RFB cells at pH=9 and a current density of 80 mA cm^−2^. e) Voltage and power density versus current density at 10, 50, and ≈100 % SOC measured in high concentration 0.4 M 2,6‐DPPAQ|1 M K_4_Fe(CN)_6_ RFB cells configured with AO‐PIM‐1 membranes. g) Photographs showing AO‐PIM‐1 and AO‐PIM‐DBMP membranes after long‐term cycling tests.

For RFBs to mature as a deployable grid storage technology, their longevity should be enhanced which largely relies on the compatibility of membrane separators and redox‐active components.[^1b^, ^3^] Firstly, to alleviate the adsorption of redox‐active molecules, a larger anthraquinone‐based redox‐active molecule with long phosphonate‐functionalized aliphatic pendants (2,6‐DPPAQ) was used as the negative electrolyte; secondly, electrolyte pH was reduced to the near neutral range so as to minimize degradation of redox‐active molecules. AO‐PIM membranes showed higher area‐specific resistance and higher voltage gap in polarization curves than those of Nafion 212, presumably due to the partial dissociation of AO functionality at pH=9, however, those values are comparable to commercial Nafion 115 membranes with the thickness being three‐time as thick as AO‐PIM membranes (Figure 5a, b and Figure S27). Encouragingly, RFBs assembled with the redox couple 2,6‐DPPAQ|K_4_Fe(CN)_6_ using AO‐PIM‐1 and AO‐PIM‐DBMP membranes delivered significantly improved cycling stability with capacity retention of 98.08 and 98.78 %, respectively, over 1000 cycles at 80 mA cm^−2^, which are superior to those obtained from the equivalent RFBs with both Nafion 212 and Nafion 115 membranes (retentions of 76.20 % and 93.87 %, respectively, Figure 5c). Impressively, the AO‐PIM‐1 cell exhibited 96.3 % of the theoretical capacity after more than 8000 cycles (29.1 days) at 80 mA cm^−2^, indicating a capacity decay rate of only 0.00045 % per cycle (0. 13 % per day, Figure 5d). Additionally, the low internal resistance of the AO‐PIM‐1 membrane enabled RFB operation at high electrolyte concentration providing significantly improved peak power density from 73 mW cm^−2^ to 240 mW cm^−2^ at ≈100 % state of charge (SOC, Figure 5e and Figure S27b). Similarly, stable cycling performance was also found for the identical cell based on AO‐PIM‐DBMP membrane showing 96.6 % of theoretical capacity after over 6000 cycles (21.7 days), equivalent to a capacity decay rate of 0.00056 % (0.16 % per day, Figure 5f) and the cell based on AO‐PIM‐SBF membrane (Figure S28). These temporal decay rates are very low compared to those reported in the literature.[^19^, ^24^] We attribute the long‐term stability of RFBs using AO‐PIM membranes to the reduced crossover, the alleviation of redox molecule adsorption, as well as improved redox molecule stability at near neutral pH. The generic application of size‐sieving AO‐PIM membranes in neutral pH RFBs was also demonstrated with another redox couple, bis(3‐trimethylammonio)propyl viologen tetrachloride and bis((3‐trimethylammonio)propyl)ferrocene (Figure S29).

## Conclusion

Ion‐conductive microporous membranes from AO‐PIMs are efficient polymer sieves that enable the long‐life cycling of aqueous organic RFBs. AO functionalization allows the maintenance of polymer rigidity and high free volume and also introduces ionizable sites for efficient ion transport, while variations of polymer chain structure enable precise control over the geometry and size of ion transport pathways. For the operation of 2,6‐DHAQ|K_4_Fe(CN)_6_ alkaline aqueous RFBs, AO‐PIM membranes exhibit low area‐specific resistance, high power density and superior energy efficiency. The durability issue induced by 2,6‐DHAQ adsorption can be circumvented by using the larger 2,6‐DPPAQ redox‐active molecule to take advantage of the excellent size‐sieving property of the AO‐PIM membranes. The 2,6‐DPPAQ|K_4_Fe(CN)_6_ aqueous RFBs assembled with AO‐PIM membranes demonstrate low temporal decay rate and high long‐term stability. Further development of the aqueous organic RFBs will also depend on the improvement of the redox molecules with high chemical stability, low decomposition rate, and high solubility in electrolytes.^[25]^

Despite the significantly enhanced cycling stability, the ion‐exchanged AO‐PIM membranes show relatively high resistance in near neutral pH electrolytes, which is mainly attributed to the relatively weak dissociation of the amidoxime groups. In principle, membrane resistance can be reduced in thin film composite (TFC) membranes. PIMs can also be combined with alternative ion‐exchange chemistries (e.g., sulfonate groups) to achieve high ionic conductivity at near‐neutral pH.^[26]^ The current methods of synthesis process make it challenging to scale and implement these PIM‐based membranes in commercial processes. Further synthetic efforts should focus both on broadening the structural diversity of PIMs to optimize performance whilst offering similar synthetic accessibility as that of AO‐PIM‐1. Such cost‐effective PIM membranes with fast and selective ion transport may fulfil their technological potential in a variety of energy‐related devices and water purification processes.

## Experimental Section

Detailed Experimental Procedures and data are provided in the Supporting Information.

## Author Contributions

Q.S. and N.B.M. conceived and supervised the project. C.Y. synthesized and characterized membranes. A.W. carried out characterizations of membranes. R.T. performed RFB tests. J.C., B.C.G., and C.G.B helped with polymer synthesis. A. A‐F, and S. G. performed SAXS/WAXS measurements and data analysis, Z. F. and J.W. helped with experiments. C.B., and K.E.J. performed modelling. N.P.B and A.R.K participated in the discussion of results. Q.S., N.B.M., C.Y., and A.W. wrote the manuscript with contributions from all authors. All authors discussed the results and commented on the manuscript at all stages.

## Conflict of interest

The authors declare no conflict of interest.

1

## Supporting information

As a service to our authors and readers, this journal provides supporting information supplied by the authors. Such materials are peer reviewed and may be re‐organized for online delivery, but are not copy‐edited or typeset. Technical support issues arising from supporting information (other than missing files) should be addressed to the authors.

Supporting Information

## Data Availability

The data that support the findings of this study are presented in the paper and Supporting Information and available from the corresponding author upon reasonable request.
