# Supplementary material for: Long‐Life Aqueous Organic Redox Flow Batteries Enabled by Amidoxime‐Functionalized Ion‐Selective Polymer Membranes
Source: Angew Chem Int Ed Engl. 2022 Aug 9;61(38):e202207580. doi: 10.1002/anie.202207580 (PMC9541571; doi:10.1002/anie.202207580)
Supplement: Supplementary file 1 — Supporting Information [file ANIE-61-0-s001.pdf]

Angewandte Chemie
Eine Zeitschrift der Gesellschaft Deutscher Chemiker

Supporting Information

Long-Life Aqueous Organic Redox Flow Batteries Enabled by Amidoxime-Functionalized Ion-Selective Polymer Membranes

C. Ye, R. Tan, A. Wang, J. Chen, B. Comesaña Gándara, C. Breakwell, A. Alvarez-Fernandez, Z. Fan, J. Weng, C. G. Bezzu, S. Guldin, N. P. Brandon, A. R. Kucernak, K. E. Jelfs, N. B. McKeown, Q. Song**

Supporting Information

Long-life Aqueous Organic Redox Flow Batteries Enabled by Amidoxime-Functionalized Ion-Selective Polymer Membranes

Chunchun Ye, Rui Tan, Anqi Wang, Jie Chen, Bibiana Comesaña Gándara, Charlotte Breakwell, Alberto Alvarez-Fernandez, Zhiyu Fan, Jiaqi Weng, C. Grazia Bezzu, Stefan Guldin, Nigel P. Brandon, Anthony R. Kucernak, Kim E. Jelfs, Neil B. McKeown, Qilei Song**

DOI: 10.1002/anie.202207580

Experimental Procedures

1. Materials and Methods

Nafion® 212 and 115 membranes (Dupont) were supplied from Sigma-Aldrich. Commercially available chemicals were used without further purification. All reactions using air/moisture sensitive reagents were performed in oven-dried apparatus under a nitrogen atmosphere.

2. AO-PIM Synthesis

General procedure (GP) for the AO functionalisation based on PIMs

Parent PIMs including PIM-1[1], PIM-SBF[2], PIM-BTrip[3] and PIM-DBMP were synthesised following previously reported methods. The amidoxime (AO) functionalisation of dibenzodioxin-based PIMs (including AO-PIM-1, AO-PIM-SBF, AO-PIM-BTrip and AO-PIM-DBMP) was conducted based on a modified procedure reported by Yavuz *et al.*[4]. The parent PIM powder was dissolved/suspended in the corresponding solvent at 70 °C under a nitrogen atmosphere. Hydroxylamine solution (50 wt. % in H_2O) was injected dropwise and the mixture was stirred at 70 °C for 24-72 h depending on the structure of PIMs, after which time a hazy solution was formed. On cooling, the hazy reaction solution was poured into ethanol to form a white precipitate. The crude product was collected by filtration, washed thoroughly with ethanol, refluxed in methanol overnight, and finally dried under *vacuum* at 110 °C for 12 h to yield AO-PIM polymer as a fine powder.

Synthesis of AO-PIM-1

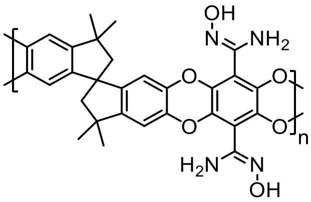

GP was followed using PIM-1 powder (1.00 g, 2.17 mmol), hydroxylamine solution (10 mL, 10.08 g), and THF (60 mL) and reacted for 24 h to afford the AO-PIM-1 polymer as a white-off powder (1.11g, 97%). ν_{max} (cm^{-1}): 3480, 3332, 3170, 2952, 1645, 1432, 1309, 998,915; ^{13}C NMR (151 MHz, solid state) δ (ppm): 164.9, 149.7, 146.3, 142.4, 138.1, 113.2, 59.4, 44.8, 32.2; GPC (DMF) Mn = 118.1 kDa, Mw = 205.2 kDa, PDI = 1.74; SA_{BET} = 567 m^2 g^{-1}, total pore volume = 0.63 cm^3 g^{-1} at P/P_0 = 0.99 from N_2 adsorption at 77K; TGA analysis: peak degradation temperature for the mass loss of the AO group decomposition is 287 °C.

Synthesis of AO-PIM-SBF

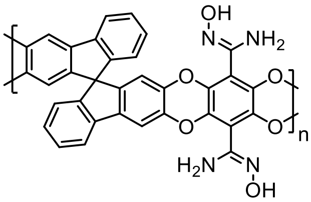

GP was followed using PIM-SBF powder (1.00 g, 2.00 mmol), hydroxylamine solution (10 mL, 10.08 g), and dimethyl sulfoxide (DMSO, 60 mL) and reacted for 48 h to afford the AO-PIM-SBF polymer as a pale-yellow powder (1.08g, 95%). ν_{max} (cm^{-1}): 3480, 3332, 3170, 1645, 1599, 1431, 1350, 1273, 1201, 1161, 1005, 984, 915, 872, 750; ^{13}C NMR (151 MHz, solid state): δ (ppm) 163.2, 145.7, 140.4, 136.6, 127.6, 122.5, 120.4, 110.6, 108.7, 65.5; GPC (DMF) Mn = 81.8 kDa, Mw = 170.2 kDa, PDI = 2.08; SA_{BET} = 611 m^2 g^{-1}, total pore volume = 0.330 cm^3 g^{-1} at P/P_0 = 0.99 from N_2 adsorption at 77K; TGA analysis: peak degradation temperature for the mass loss of the AO group decomposition is 260 °C.

Synthesis of AO-PIM-BTrip

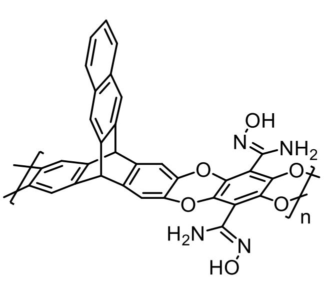

GP was followed using PIM-BTrip powder (1.00 g, 1.94 mmol), hydroxylamine solution (10 mL, 10.08 g), and dimethyl sulfoxide (DMSO, 60 mL) and reacted for 72 h to afford the AO-PIM-BTrip polymer as a pale-yellow powder (1.04 g, 92%). ν_{max} (cm^{-1}): 3480, 3332, 3170,

1645, 1424, 1274, 991, 915, 887, 756; ^{13}C NMR (151 MHz, solid state): δ (ppm) 163.5, 144.7, 138.4, 131.6, 126.4, 118.3, 110.8, 47.6, 13.1; GPC (DMF) Mn = 74.9 kDa, Mw = 145.7 kDa, PDI = 1.94; SA_{BET} = 649 $m^2\ g^{-1}$, total pore volume = 0.350 $cm^3\ g^{-1}$ at P/P_0 = 0.99 from N_2 adsorption at 77K; TGA analysis: peak degradation temperature for the mass loss of the AO group decomposition is 279 °C.

Synthesis of AO-PIM-DBMP

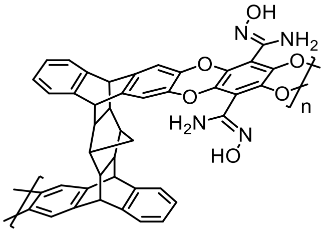

GP was followed using PIM-DBMP powder (1.00 g, 1.58 mmol), hydroxylamine solution (10 mL, 10.08 g), and *N*-methyl-2-pyrrolidone (NMP, 60 mL) and reacted for 72 h to afford the AO-PIM-DBMP polymer as a pale-yellow powder (0.99 g, 90%). ν_{max} (cm^{-1}): 3480, 3332, 3170, 2930, 1645, 1430, 1285, 1145, 995, 915, 742; ^{13}C NMR (151 MHz, solid state) δ (ppm) 163.3, 140.4, 125.0, 112.5, 58.4, 48.8, 44.0, 26.9; GPC (DMF) Mn = 38.6 kDa, Mw = 99.1 kDa, PDI = 2.04; SA_{BET} = 608 $m^2\ g^{-1}$, total pore volume = 0.407 $cm^3\ g^{-1}$ at P/P_0 = 0.99 from N_2 adsorption at 77K; TGA analysis: peak degradation temperature for the mass loss of the AO group decomposition is 276 °C.

3. Membrane Formation

The AO-PIM polymer was dissolved (~ 0.23 g) in appropriate polar aprotic solvents (15-20 mL) depending on its solubility and viscosity (*i.e.,* DMF for AO-PIM-1, DMSO for AO-PIM-SBF and AO-PIM-BTrip, and NMP for AO-PIM-DBMP, respectively) to form a clear casting solution. The polymer solution was centrifuged (12,000 rpm for 5 min) to remove insoluble impurities and then cast on a clean glass petri dish (diameter = 9 cm). The AO-PIM membrane was allowed to form by slow solvent evaporation in an oven at 65 °C for 48-72 h. Dry 50-μm-thick membrane was peeled off from the petri dish by immersing in deionized water, and cut into needed sizes for further measurements.

4. Characterization Techniques

Fourier transform Infrared Spectroscopy (FT-IR). FT-IR spectra of polymer powder or membrane samples were obtained by a Perkin-Elmer 1600 FTIR spectrometer with absorptions in the range 4000-500 cm^{-1}.

Nuclear Magnetic Resonance (NMR). ^{13}C solid-state NMR spectra of AO-PIM powders were collected using a Bruker AVA 600 spectrometer with a spinning rate of 12,000 Hz and an adamantane reference. Spectra were compiled from 6,000 scan numbers with a 6 s recycle delay.

Gel permeation chromatography (GPC). GPC curves of AO-PIM polymers were analysed by a GPC Agilent 1260 system equipped with two PLgel MIXED-C columns and a RI detector. Samples were dissolved in DMF containing 0.1% w/v lithium bromide (~ 5 mg mL^{-1}) and operated at a flow rate of 1 mL min^{-1} and 60 °C. Molecular weight and polydispersity index (PDI) was calculated based on poly(methyl methacrylate) standards.

Thermogravimetric analysis (TGA). TGA traces of polymer powder were obtained by a NETZSCH STA 449 F5 Jupiter thermogravimetric analyser at a heating rate of 10 °C min^{-1} from room temperature to 800 °C under a nitrogen atmosphere.

Skeletal density test. Skeletal density test was performed by a Micromeritics Accupyc II 1340 helium pycnometer with a 3.5 cm^3 sample chamber at 25 °C. AO-PIM powders were degassed overnight at 110 °C under *vacuum* prior to tests. The mean values and standard deviation were calculated based on 10 measurements.

Tensile test. Tensile test was conducted on a Lloyd-Ametek EZ50 Material Testing Machine at ~50% relative humidity and room temperature. Membrane specimens with the width of ~5 mm and effective length of ~30 mm were strained at a rate of 3 mm min^{-1}. Average values and standard deviation were calculated on 3-5 specimens.

Nitrogen/carbon dioxide physisorption. Low-pressure gas physisorption were measured by a Micromeritics 3Flex surface characterization analyser with N_2 isotherms at 77 K and CO_2 physisorption at 273 K. Powder samples (~100 mg) were degassed at 110 °C under high *vacuum* for 12 h followed by loading into the instrument and *in situ* degassed at 110 °C for another 12 h prior to measurements.

Dynamic water vapour sorption (DVS). Dynamic water vapour sorption was measured on a dynamic vapour sorption Endeavour gravimetric sorption analyser (Surface Measurement Systems Ltd.) with the relative humidity in the range of 0 - 90%. Membrane samples (20-30 mg) were dried at 110 °C under *vacuum* for 12 h followed by loading into the instrument and *in situ* dried at 25 °C under flowing dry air over 24 h until the mass keeping constant. Water vapour uptake isotherms were obtained by measuring sample mass change in dry to fully hydrated state at a specified relative humidity.

Small-angle X-ray scattering (SAXS)/wide-angle X-ray scattering (WAXS). SAXS and WAXS experiments were carried out on a Ganesha 300XL (SAXSLAB) instrument at the EPSRC CNIE research facility at University College London by employing a high brilliance microfocus Cu source (λ = 1.54 Å). SAXS and WAXS patterns were recorded using a Pilatus 300 K solid-state photon-counting detector with a 2 mm beam stop with a sample-to-detector distance of 1041 mm (SAXS) and of 101 mm (WAXS), respectively. The

beam center and the sample-to-detector distance were calibrated using the position of diffraction peaks from a standard silver behenate powder. SAXS and WAXS patterns were radially averaged around the direct beam position using SAXSGUI software.

5. Experimental Measurements

Water/electrolyte uptake measurement

Water/electrolyte uptake was measured by mass changes of AO-PIM membrane samples in dry and fully hydrated conditions. Membrane samples were dried at 110 °C under *vacuum* overnight to obtain the dry mass (W_{dry}). These samples were immersed in deionized water, 1M aqueous KOH and 1M aqueous KCl in sequence by using the same membranes for 24 h at room temperature. Before changing the liquid, the wet mass of membranes was measured immediately after wiping off excess liquid from the surface (W_{wet}). The water or aqueous electrolyte uptake (WU/EU) was calculated by:

$$WU / EU(\%) = \frac{W_{wet} - W_{dry}}{W_{dry}} \times 100\% \qquad \text{(Equation S1)}$$

The mean values and error bars are based on 3 measurements using 3 different membrane samples.

Swelling ratio measurement

The swelling ratio (SR) was calculated from the difference in linear dimensions between the wet (x_{wet}) and dry (x_{dry}) AO-PIM membranes according to:

$$SR(\%) = \frac{x_{wet} - x_{dry}}{x_{dry}} \times 100\% \qquad \text{(Equation S2)}$$

Error bars are standard deviations derived from 3 measurements based on 3 different membrane specimens.

Ionic conductivity measurement

Ionic conductivity of AO-PIM membranes was performed on electrochemical impedance spectroscopy (EIS) using the potentiostat mode at an AC bias of 10 mV and a frequency range from 0.2 MHz to 10 Hz. For apparent ionic conductivity tests, membrane samples were pretreated in 1M aqueous KOH overnight to fully deprotonate hydroxyl groups (*i.e.,* to convert -OH form into $-O^-$ form), followed by soaking in deionized H_2O for 24 h to remove residual KOH, then equilibrated in deionized H_2O, 1M KOH or 1M KCl aqueous electrolyte (termed as ^{a}KCl conductivity) for 24 h. Membrane samples in non-deprotonated form (*i.e.,* -OH form) were directly soaked in 1M aqueous KCl for 24 h for the intrinsic ionic conductivity tests (termed as ^{b}KCl conductivity). Membrane samples were sandwiched between two stainless steel electrodes and sealed with coin cells (Type 2032). The assembly procedure was carried out in deionized H_2O or KCl/KOH electrolytes to avoid air bubbles being trapped in cells. The ionic conductivity in the range of 30 to 80 °C was calculated from the resistance[5] by:

$$\sigma = \frac{L}{AR_m} \qquad \text{(Equation S3)}$$

where σ is the membrane ionic conductivity in S cm^{-1}, L is the membrane thickness in cm, A is the membrane active area of 2.00 cm^2, and R_m is the membrane resistance in Ω. The resistance of blank coin cell (0.04 Ω) was subtracted from R_m before applying Equation S3.

Diffusion and Crossover measurement

Ion diffusion and redox molecule crossover tests were performed using concentration-driven dialysis diffusion H-shaped cells. AO-PIM membranes were sandwiched between two chemically resistant O-rings and secured in the middle of H-cells by clips. Continuous stirring was used in both feed and permeate sides to alleviate the concentration polarization near membranes.

In ion diffusion dialysis tests, 50-μm-thick AO-PIM membranes in non-deprotonation, neutral-charge form (*i.e.,* -OH) were first applied to evaluate the permeation rates of common salts to investigate their ion-sieving performance. Aqueous electrolytes (*i.e.,* 1 M KCl, NaCl, LiCl, $CaCl_2$ and $MgCl_2$, 50 mL) was used as a feed solution and deionized H_2O (50 mL) was used in the permeate side. Besides, AO-PIM membranes in deprotonation, negative-charge form (*i.e.,* $-O^-$) were then used to measure the permeation rates of KOH (1M, 50 mL, pH = 14.0) and KCl (1M, 50 mL, pH = 9.0) across the membranes. The ionic conductivity in the permeate side was continuously recorded by a conductivity meter (Orion Star A210, Thermo Scientific). The concentration change of aqueous electrolytes over time was obtained from the corresponding electrolyte calibration curves according to the linear relationship between the ionic conductivity and electrolyte concentration. The mean values and error bars of ion permeation rate were derived from 3 individual measurements based on 3 different samples.

In redox molecule crossover tests, AO-PIM membranes were pretreated in 1M KOH to obtain their deprotonated form. In alkaline system, $K_4Fe(CN)_6$ (0.1M) or 2,6-DHAQ (0.1M) in aqueous KOH solution (1M, 50 mL, pH = 14.0) was used as a feed solution and blank aqueous KOH solution (1M, 50 mL, pH = 14.0) was used in the permeate side. In near neutral-pH system, $K_4Fe(CN)_6$ (0.1M) or 2,6-DPPAQ (0.1M) in KCl aqueous solution (1M, 50 mL, pH = 9.0) was used as a feed solution and blank KCl aqueous solution (1M, 50 mL, pH = 9.0) was used in the permeate side. The concentration change of $K_4Fe(CN)_6$ in the permeate side was quantitatively detected by inductively coupled plasma-optical emission spectrometry (ICP-OES). Permeate aliquots were collected three times per day and diluted with 2.0 wt.% HNO_3 (100 or 50 times) prior to analysis. The concentration changes of 2,6-DHAQ and 2,6-DPPAQ in the permeate

side was quantitatively detected by the calibrated UV-Vis spectrometer. Permeate aliquots were analysed without dilution and recycled to permeate side.

The permeation rate of ions and redox molecules across the membrane over a short-time period without volume change can be determined by Fick's first law:

$$J = \frac{V}{A}\left(\frac{\partial C}{\partial t}\right) \quad \text{(Equation S4)}$$

where J is the permeation rate in mol cm^{-2} s^{-1}, V is the solution volume of 50 mL, A is the membrane active area of 1.54 cm^2, C is permeate concentration in mol cm^{-3}, and t is diffusion time in s. Over the process, the difference between permeate and feed concentration can be ignored (*i.e.,* $C_1 - C_2 = C_1$), and the flux can be assumed to be constant. Consequently, Fick's first law can be simplified as:

$$J = \frac{D(C_1 - C_2)}{l} = \frac{DC_1}{l} \quad \text{(Equation S5)}$$

where D is the diffusivity in cm^2 s^{-1}, C_1 is feed concentration in mol cm^{-3}, and l is the membrane thickness in cm.

Flow battery measurement

The flow cell was assembled with a cell fixture (Scribner Associates) and POCO single serpentine pattern graphite plates. A piece of ~50-μm-thick membrane (active area = 7 cm^2) was sandwiched between electrodes comprising a stack of carbon paper (three sheets, SGL) in each side. The rest space between graphite plates was sealed by Viton gasket. Electrolytes were fed into the cell at a flow rate of 100 mL min^{-1} by a Cole-Parmer peristaltic pump.

Carbon paper was pretreated by baking at 400 °C in air for 24 h. Nafion® 212 and 115 membranes were pretreated by soaking in deionized water at 80 °C for 20 min, followed in hydrogen peroxide solution (6%) at room temperature for 35 min, and stored in 0.1M aqueous KCl solution at room temperature for further tests. AO-PIM membranes were pretreated in 1M KOH for 24 h to obtain their deprotonated form. Prior to full cell tests, Nafion® and AO-PIM membranes were equilibrated in 1M aqueous KOH (for alkaline flow cell tests) or 1M aqueous KCl with pH = 9 (for near neutral-pH flow cell tests) overnight. For alkaline 2,6-DHAQ|$K_4Fe(CN)_6$ flow cell tests, the electrolytes consisted of 0.1M $K_4Fe(CN)_6$, or 0.1M 2,6-DHAQ combined with 0.2M KOH in 10 mL 1M aqueous KOH solution. For near neutral-pH 2,6-DPPAQ|$K_4Fe(CN)_6$ flow cell tests, the electrolytes consisted of 0.1M $K_4Fe(CN)_6$, or 0.1M 2,6-DPPAQ combined with 0.4M KOH in 10 mL 1M aqueous KCl solution. Trace amount of KOH was added to catholyte and analyte solutions to adjust the pH to 9.0. For neutral-pH BTMAP-Vi|BTMAP-Fc flow cell tests, the electrolytes consisted of 0.1M BTMAP-Vi or 0.1M BTMAP-Fc in 10 mL 1 M aqueous NaCl solution.

EIS spectra at 0 and ~100% state of charge (SOC) was measured using the potentiostat mode at an AC bias of 10 mV and a frequency range from 0.5 MHz to 10 Hz in the assembled flow cells. Charging-discharging curves were obtained using an electrochemical station (Biologic SP-150 potentiostat) with a constant current density at room temperature. To obtain an electrochemical polarization curve, the cell was charged to a desired SOC followed by polarized using linear galvanic sweep method at a rate of 200 mA s^{-1} from -6000 to 6000 mA. The corresponding power density at specific SOC (10, 50, ~100 %) was derived from the current - voltage curve. Long-term cycling tests were performed at 80 mA cm^{-2} for the 2,6-DHAQ|$K_4Fe(CN)_6$ and 2,6-DPPAQ|$K_4Fe(CN)_6$ cells and at 25 mA cm^{-2} for BTMAP-Vi|BTMAP-Fc cells in an argon-filled glove box. The coulombic efficiency (CE) was calculated by the ratio of discharge capacity to charge capacity. The voltage efficiency (VE) was calculated by the ratio of average discharge voltage to average charge voltage. The energy efficiency (EE) was calculated by the ratio of average discharge energy to average charge energy. The cyclic capacity decay rates of different membranes were evaluated by the overall percentage loss of capacity divided by the total operation time (cycle numbers or days).

6. Computational Methods

Density Functional Theory (DFT) Calculations

DFT calculations were carried out to probe the charged properties of the redox active species and AO-PIM polymer segments. Geometry optimisations of the redox active species as well as single hydrogen capped cluster models of the AO-PIM polymers were carried out using Gaussian16[6] with the Becke's 3 parameter exchange function combined with Lee–Yang–Parr correlation functional (B3LYP)[7]. The Los Alamos National Laboratory 2 Double-Zeta (LANL2DZ)[8] basis set was used for iron (Fe) atoms and the 6-31+G(d,p) basis set was used for all other atoms (H, C, N, O, P). Molecular electrostatic potential (MESP) surfaces were calculated for each optimized geometry and plotted on an isosurface of 0.002 a.u.

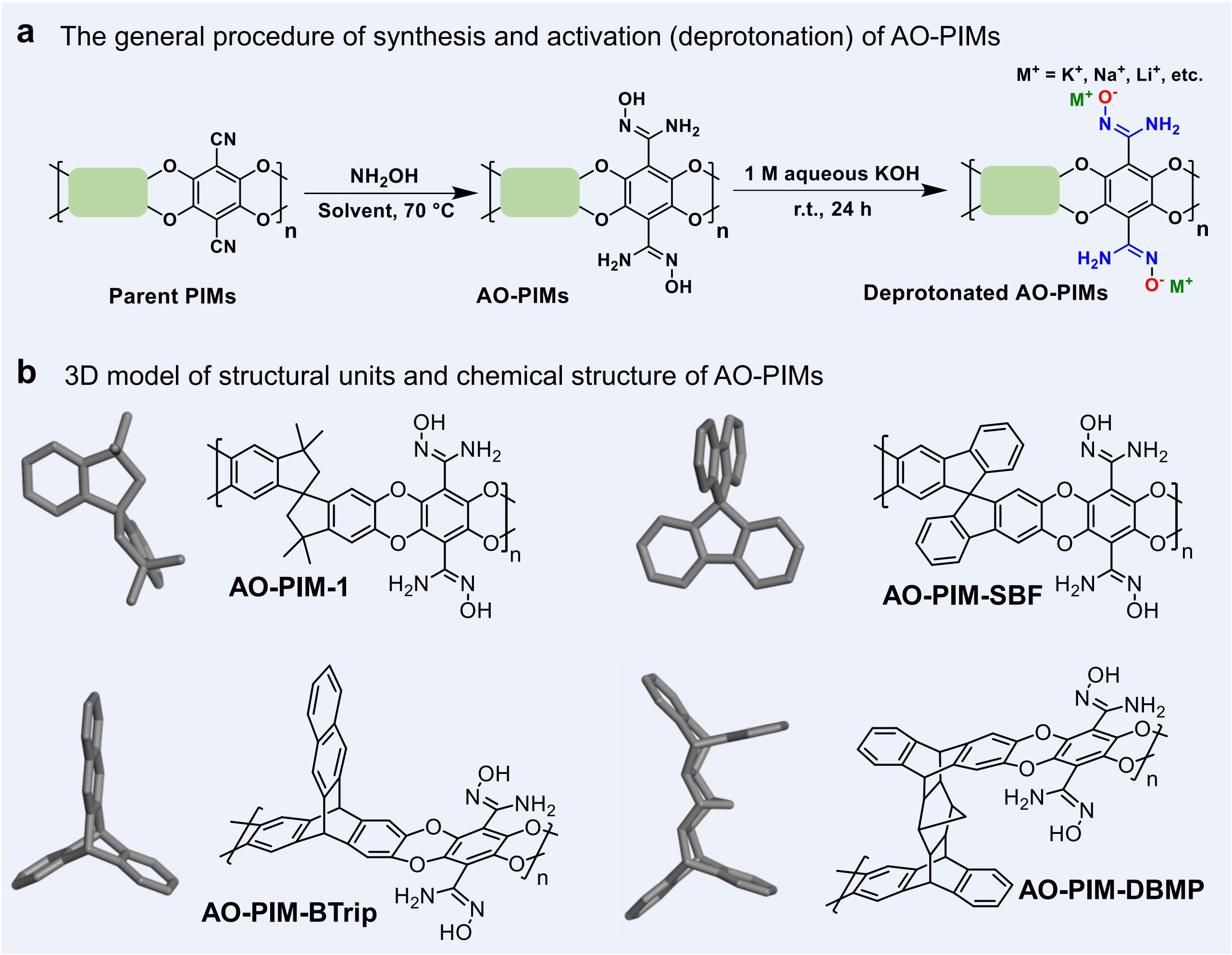

Figure S1. General synthetic route of AO-PIMs. (a) AO functionalisation and activation (deprotonation) routes of parent PIMs. (b) 3D model of structural units and chemical structures of AO-PIMs with 3D (*i.e.,* AO-PIM-1 and AO-PIM-SBF) or 2D (*i.e.,* AO-PIM-BTrip and AO-PIM-DBMP) macromolecular chains.

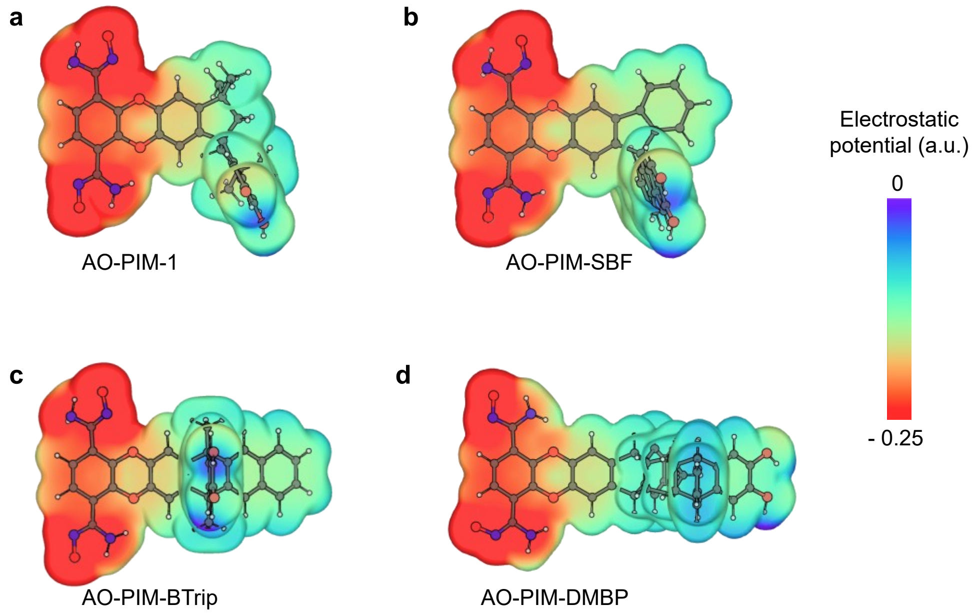

Figure S2. Electrostatic potential (ESP) of (a) AO-PIM-1 (b) AO-PIM-SBF (c) AO-PIM-BTrip and (d) AO-PIM-DBMP structural units.

Table S1. ESP of three redox couples for aqueous organic RFBs (The unit of ESP map is a.u.)

**Catholytes**	**Anolytes**
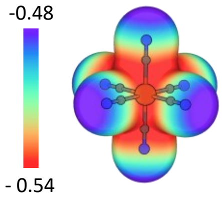 $Fe(CN_6)^{4-}$	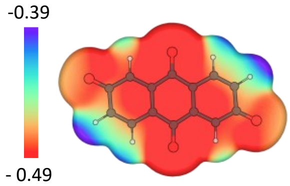 2,6-DHAQ^{4-}
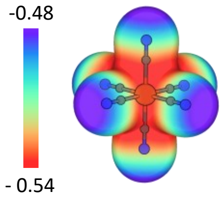 $Fe(CN_6)^{4-}$	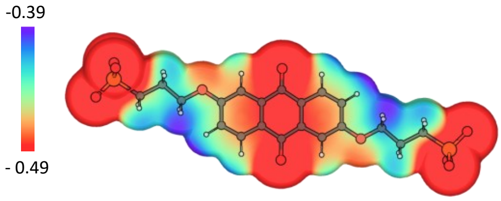 2,6-DPPAQ^{6-}
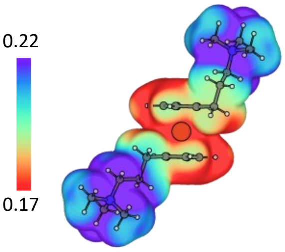 BTMAP-Fc^{2+}	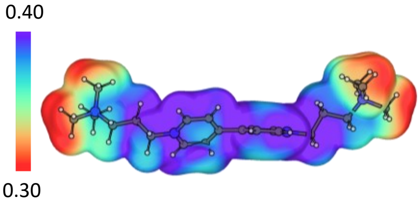 BTMAP-Vi^{4+}

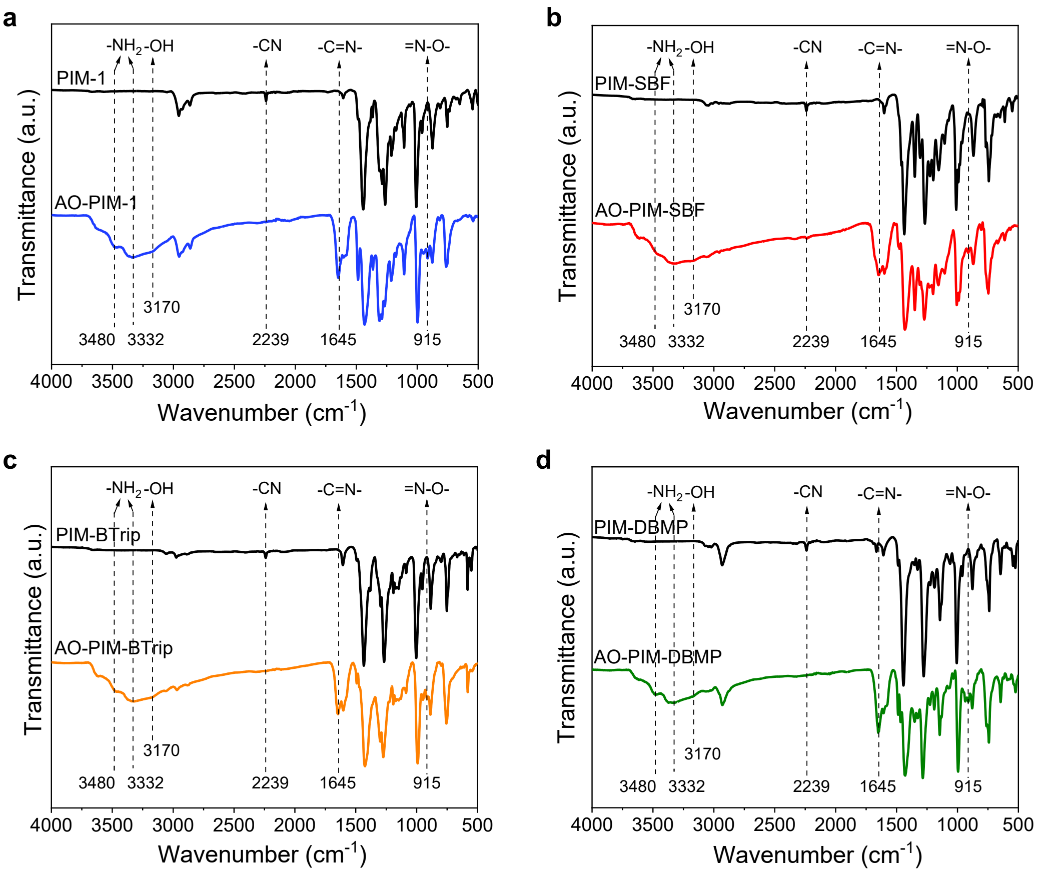

Figure S3. FTIR spectra of AO-PIM polymers. (a) AO-PIM-1, (b) AO-PIM-SBF, (c) AO-PIM-Btrip and (d) AO-PIM-DBMP. The characteristic peak appearing at 2239 cm^{-1} is assigned to the stretching band of CN groups in parent PIMs, however, no CN group peak is visible in AO-PIMs after performing AO functionalisation. Other obvious vibrations for AO groups appear including the bands at 3480 and 3332 cm^{-1} from antisymmetric and symmetric stretching of NH_2 groups, 3170 cm^{-1} from stretching vibration of OH groups, 1645 and 915 cm^{-1} from C=N and N-O stretching of oxime groups[4], respectively. These distinct peaks attributed to AO functionality indicate the complete conversion of nitrile groups to amidoxime groups in all four PIM polymers.

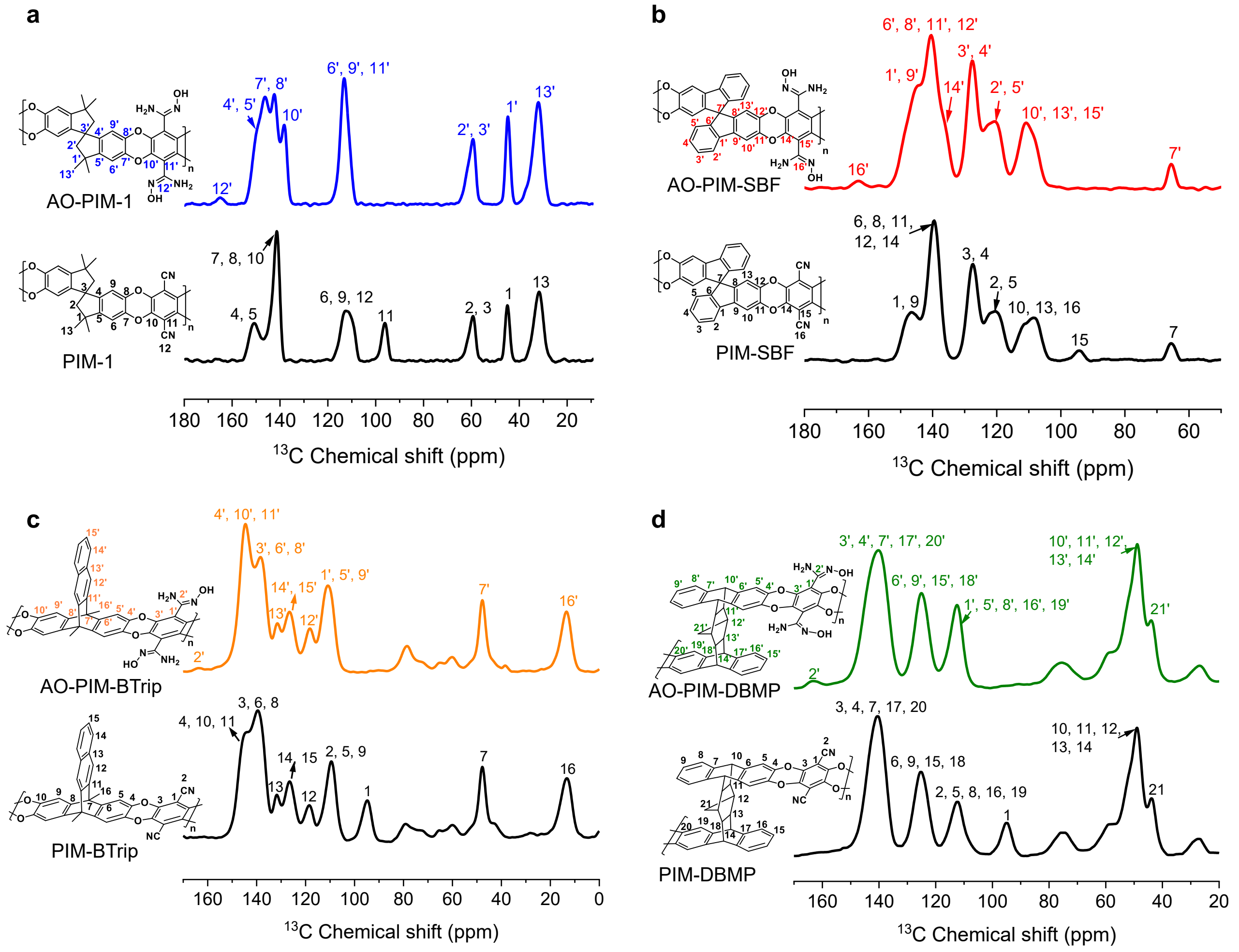

Figure S4. Chemical structure, solid-state ^{13}C NMR spectra and peak assignment of AO-PIMs. (a) PIM-1 and AO-PIM-1[5], (b) PIM-SBF and AO-PIM-SBF (c) PIM-BTrip and AO-PIM-BTrip and (d) PIM-DBMP and AO-PIM-DBMP. Chemical shift around 95 ppm exclusively corresponds to carbons adjacent to CN groups in parent PIMs, *i.e.,* 96.0 ppm is carbon (11) of PIM-1, 94.2 ppm is carbon (15) of PIM-SBF, 94.8 ppm is carbon (1) of PIM-BTrip, and 95.1 ppm is carbon (1) of PIM-DBMP. Chemical shifts around 165 ppm are carbons belong to AO groups in AO-PIMs, *i.e.,* 164.9 ppm is carbon (12') of AO-PIM-1, 163.2 ppm is carbon (16') of AO-PIM-SBF, 163.5 ppm is carbon (2') of AO-PIM-BTrip, and 163.3 ppm is carbon (2') of PIM-DBMP, respectively. The total disappearance of chemical shift adjacent to CN groups in parent PIMs, and appearance of new chemical shift for AO groups in AO-PIMs signify the complete AO functionalisation of four dibenzodioxin-based PIMs.

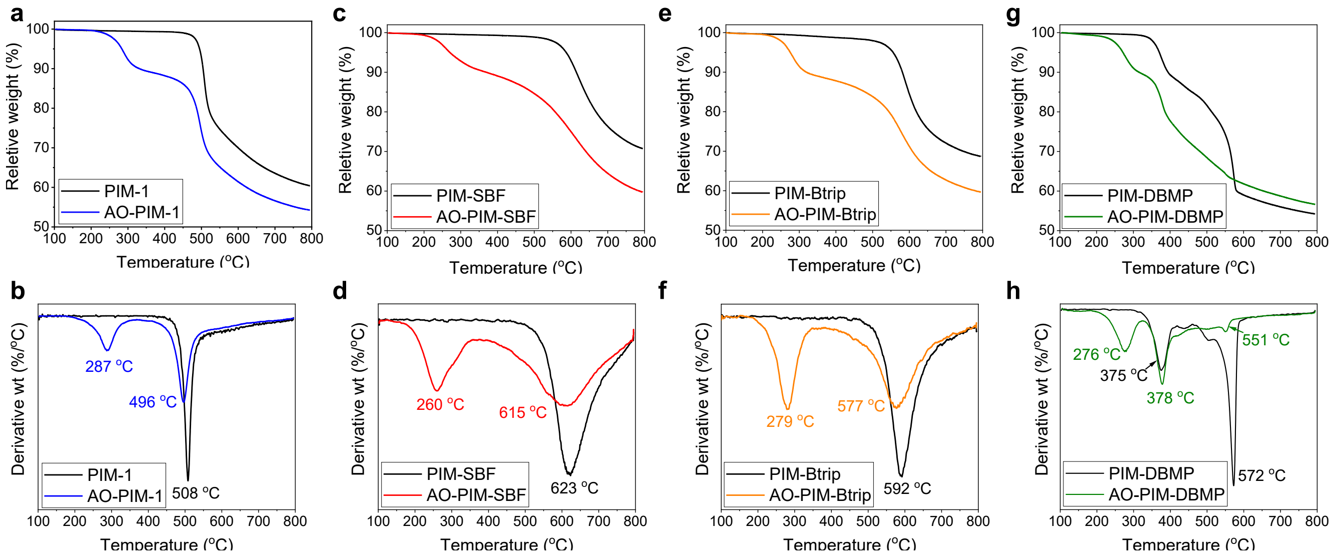

Figure S5. Thermal stability of AO-PIM polymers. TGA thermogram and differential thermo-gravimetric plots of (a) (b) AO-PIM-1, (c) (d) AO-PIM-SBF, (e) (f) AO-PIM-BTrip and (g) (h) AO-PIM-DBMP. TGA analysis shows the similar first-stage mass loss of four AO-PIM polymers in the temperature range of 260-287 °C, attributed to the degradation of AO functionalities.

Table S2. TGA results of PIM and AO-PIM polymers.

Polymers	Mass loss of AO groups (T_{d1}[a], °C)	Backbone degradation (T_{d2}[b], °C)
PIM-1	-	508
AO-PIM-1	287	496
PIM-SBF	-	623
AO-PIM-SBF	260	615
PIM-BTrip	-	592
AO-PIM-BTrip	279	577
PIM-DBMP	-	375, 572
AO-PIM-DBMP	276	378, 551

[a] T_{d1} is the peak degradation temperature for the mass loss of AO groups.
[b] T_{d2} is the peak degradation temperature for the backbone degradation.

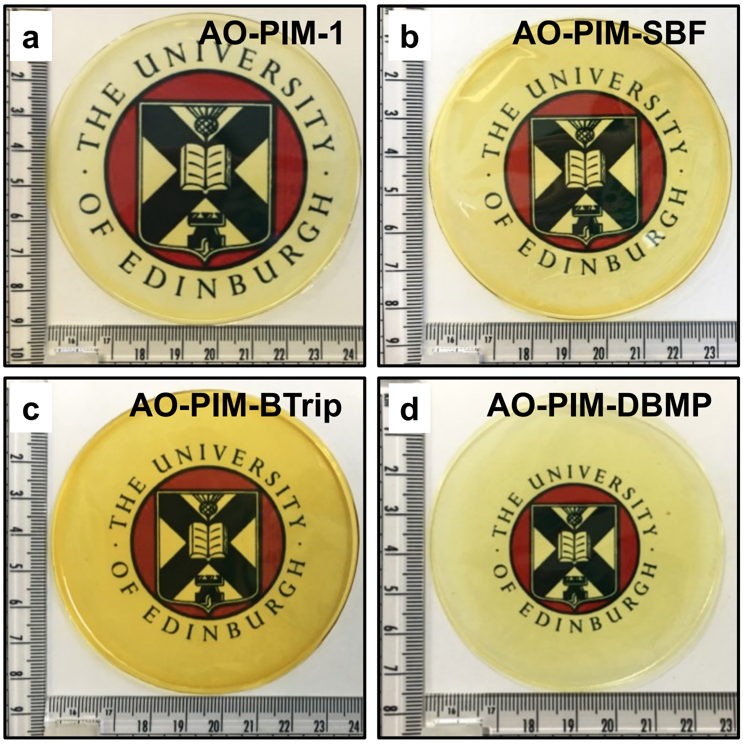

Figure S6. Photographs of AO-PIM membranes fabricated by solution-casting method.

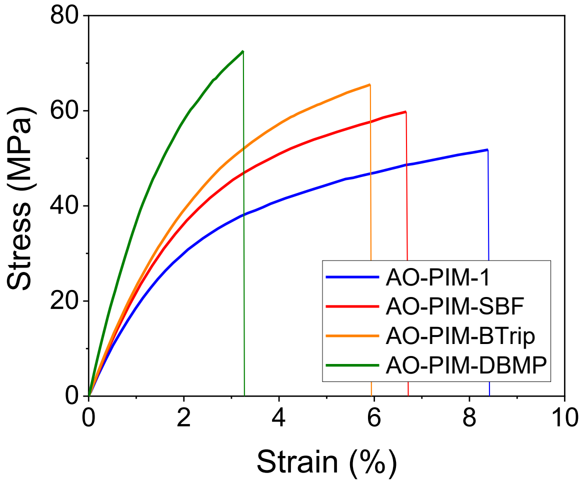

Figure S7. Stress-strain curves of AO-PIM membranes. The mechanical behaviours of AO-PIMs follow a general feature of glassy polymers, *i.e.*, exhibiting relatively high toughness and stiffness[9], which indicate their high rigidity and low mobility of PIM polymeric chains.

Table S3. Mechanical properties of AO-PIM membranes.

Polymers	Young's Modulus (MPa)	Tensile strength (MPa)	Elongation at break (%)
AO-PIM-1	1777 ± 70	51.8 ± 3.5	8.4 ± 0.3
AO-PIM-SBF	2059 ± 148	59.8 ± 4.7	6.7 ± 0.4
AO-PIM-BTrip	2245 ± 132	65.5 ± 3.9	5.9 ± 0.3
AO-PIM-DBMP	2829 ± 182	72.6 ± 5.2	3.3 ± 0.1

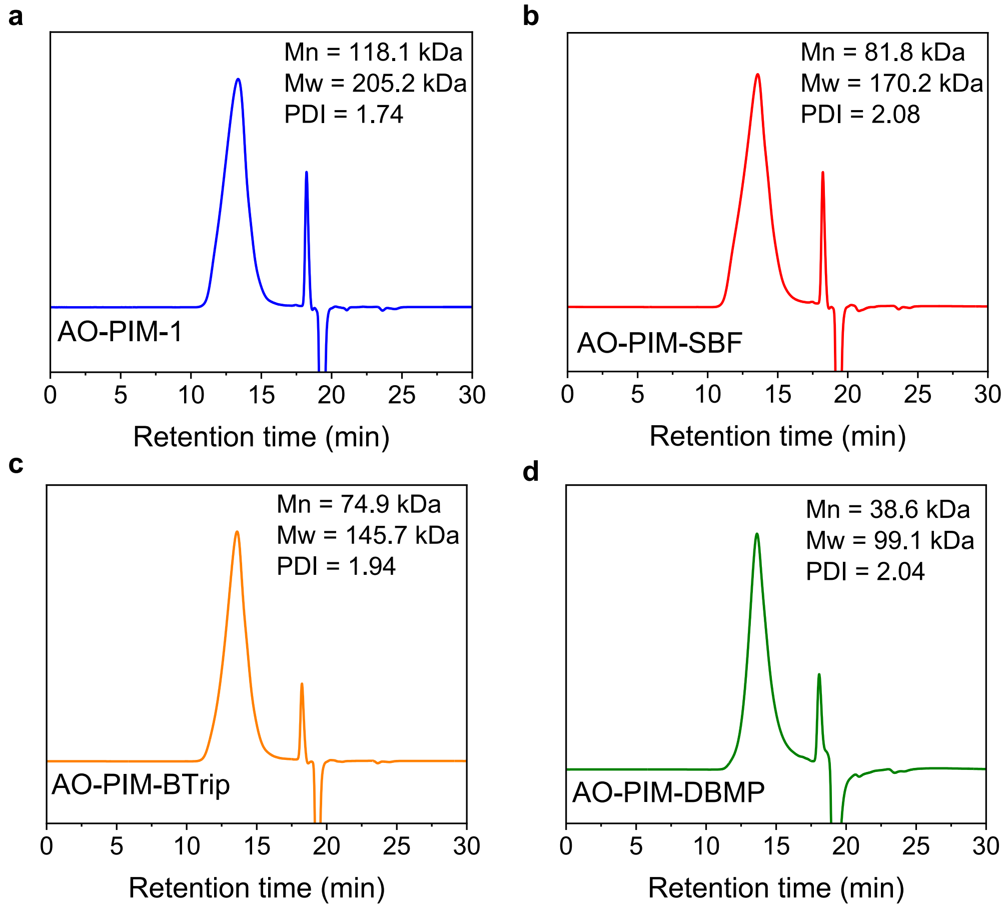

Figure S8. GPC traces of AO-PIMs. (a) AO-PIM-1, (b) AO-PIM-SBF, (c) AO-PIM-BTrip and (d) AO-PIM-DBMP.

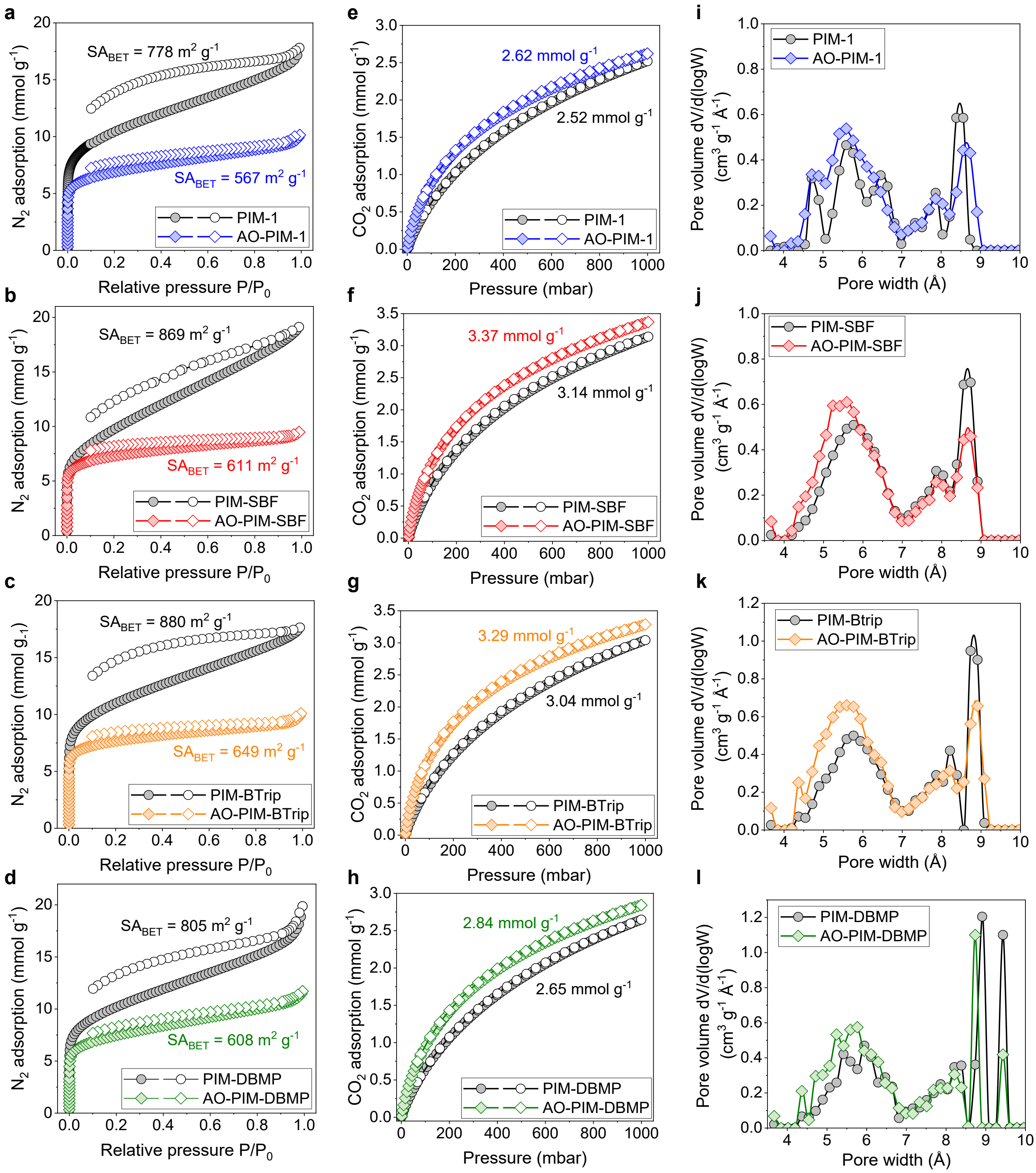

Figure S9. Gas sorption of AO-PIM polymers. N_2 adsorption-desorption isotherms at 77K, CO_2 adsorption-desorption isotherms at 273K, and derived pore size distribution based on DFT calculation of (a) (e) (i) AO-PIM-1[5], (b) (f) (j) AO-PIM-SBF, (c) (g) (k) AO-PIM-BTrip, and (d) (h) (l) AO-PIM-DBMP, respectively. Solid symbols: adsorption; open symbols: desorption.

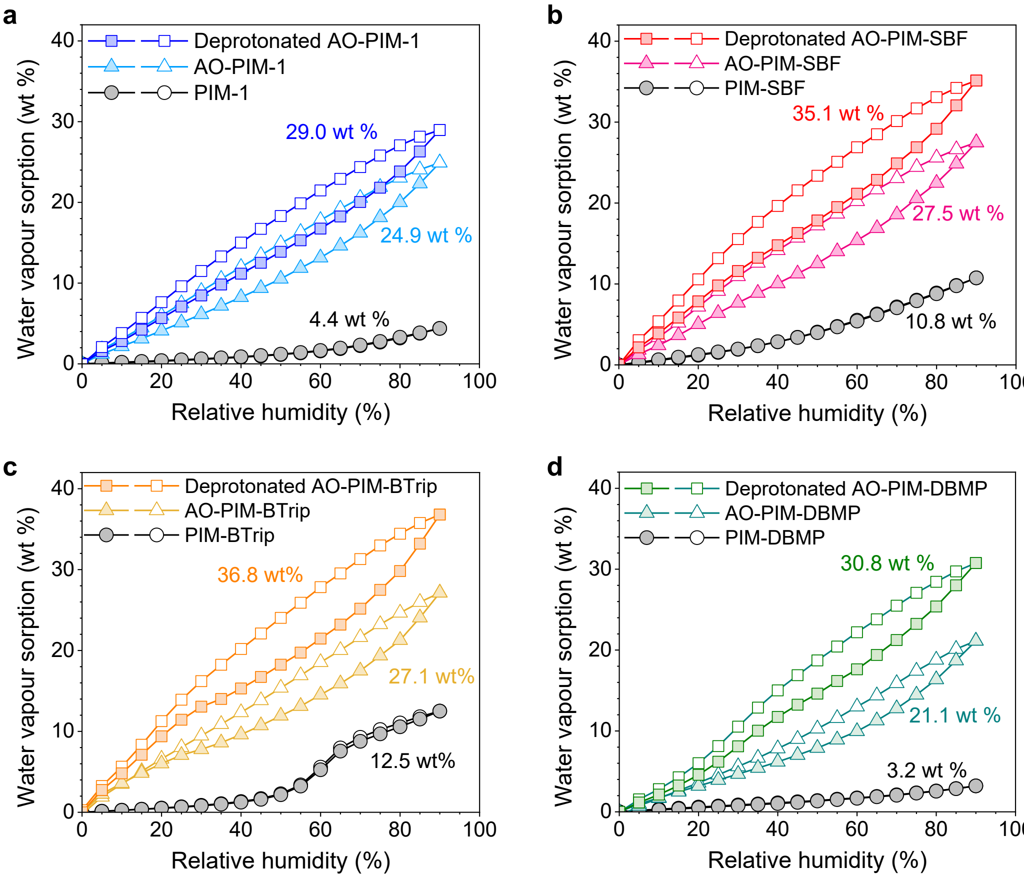

Figure S10. Water vapour uptake isotherms of AO-PIM polymers as a function of relative humidity ranging from 0 % to 90 % at 25 °C. (a) AO-PIM-1[5], (b) AO-PIM-SBF, (c) AO-PIM-BTrip, and (d) AO-PIM-DBMP membranes. Solid symbols: adsorption; open symbols: desorption.

Table S4. Physical properties of parent PIM and AO-PIM polymers.

Polymers	SA_{BET}[a] (m^2 g^{-1})	Total pore volume[b] (cm^3 g^{-1})	Skeletal density (g cm^{-3})	Envelop density (g cm^{-3})	Water vapour uptake[c] (wt.%)	Water vapour uptake[d] (wt.%)
PIM-1[e]	778	0.588	1.2339 ± 0.0011	1.043	4.4	-
AO-PIM-1[e]	567	0.353	1.2590 ± 0.0038	1.210	24.9	29.0
PIM-SBF	869	0.623	1.3269 ± 0.0044	1.091	10.8	-
AO-PIM-SBF	611	0.330	1.3447 ± 0.0020	1.240	27.5	35.1
PIM-BTrip	880	0.612	1.3370 ± 0.0021	1.153	12.5	-
AO-PIM-BTrip	649	0.350	1.3766 ± 0.0029	1.298	27.1	36.8
PIM-DBMP	805	0.690	1.2974 ± 0.0025	1.130	3.2	
AO-PIM-DBMP	608	0.407	1.3238 ± 0.0090	1.289	21.1	30.8

[a] SA_{BET} was obtained by N_2 adsorption in the range of P/P_0 = 0.001-0.1.
[b] Total pore volume was obtained from N_2 adsorption based on the NLDFT model.
[c] Water vapour uptake was measured using initial non-deprotonated AO-PIM membranes by DVS.
[d] AO-PIM membranes were soaked in 1M NaOH solution overnight to yield deprotonated forms then their water vapour uptake was obtained by DVS.
[e] The values of $SA_{BET,}$ total pore volume and water vapour uptake of PIM-1 and AO-PIM-1 were from our previously reported work[5].

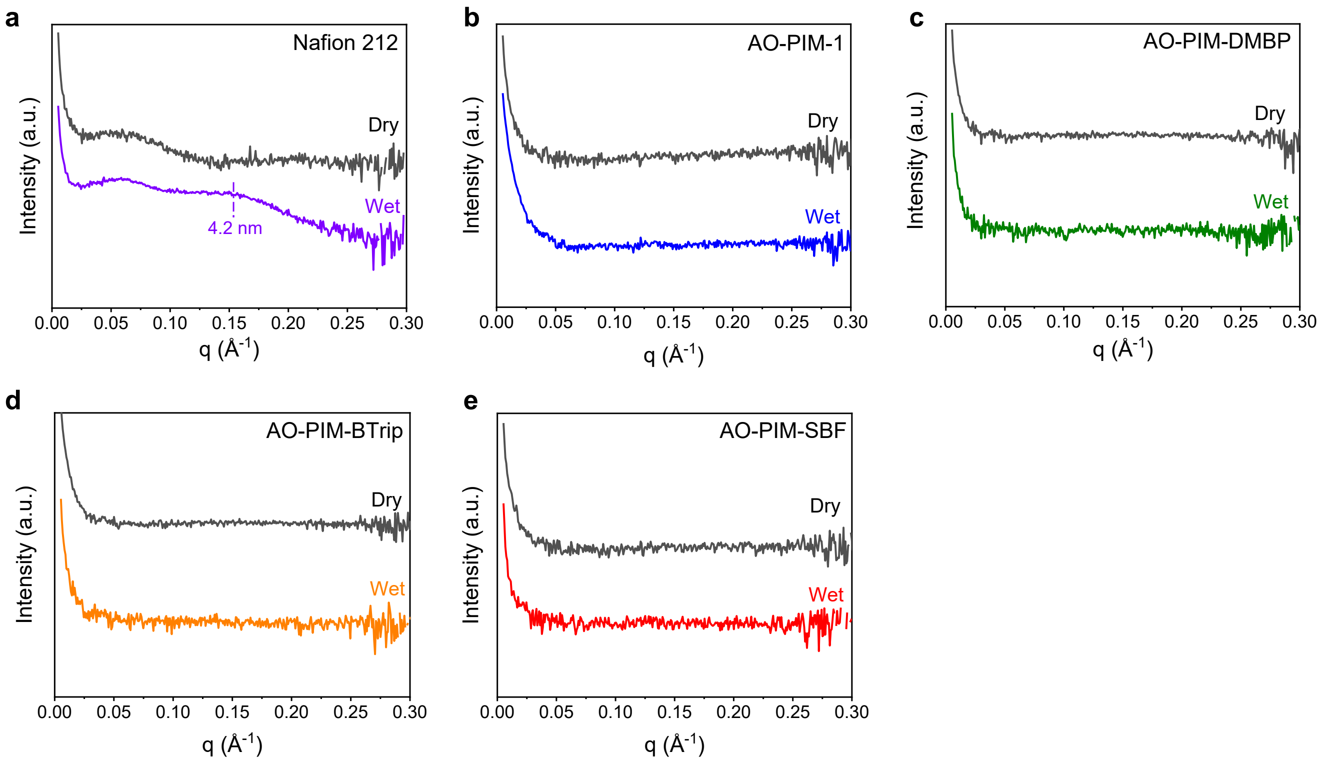

Figure S11. Small-angle X-ray scattering (SAXS) patterns of ionised membranes. (a) Nafion 212, (b) AO-PIM-1, (c) AO-PIM-DMBP, (d) AO-PIM-BTrip and (e) AO-PIM-SBF were tested before and after wetted by deionized water. Power-law scattering patterns relevant to a fractural surface structure at low q (< 0.05 $Å^{-1}$) were observed for all samples. A broad scattering feature at q ~0.15 $Å^{-1}$ (~4.2 nm in real space) was observed for wet Nafion 212 membrane, corresponding to the spacing of hydrophilic water domains in Nafion. This typical ionomer peak and nanoscale pore channel within Nafion membranes has been widely studied[10]. In contrast, all AO-PIM membranes did not present distinct scattering features over a wide low-q range from 0.05 to 0.3 $Å^{-1}$ in both dry and wet states which supports the assumption of amorphous homopolymers without large intersegmental volume and local phase separation.

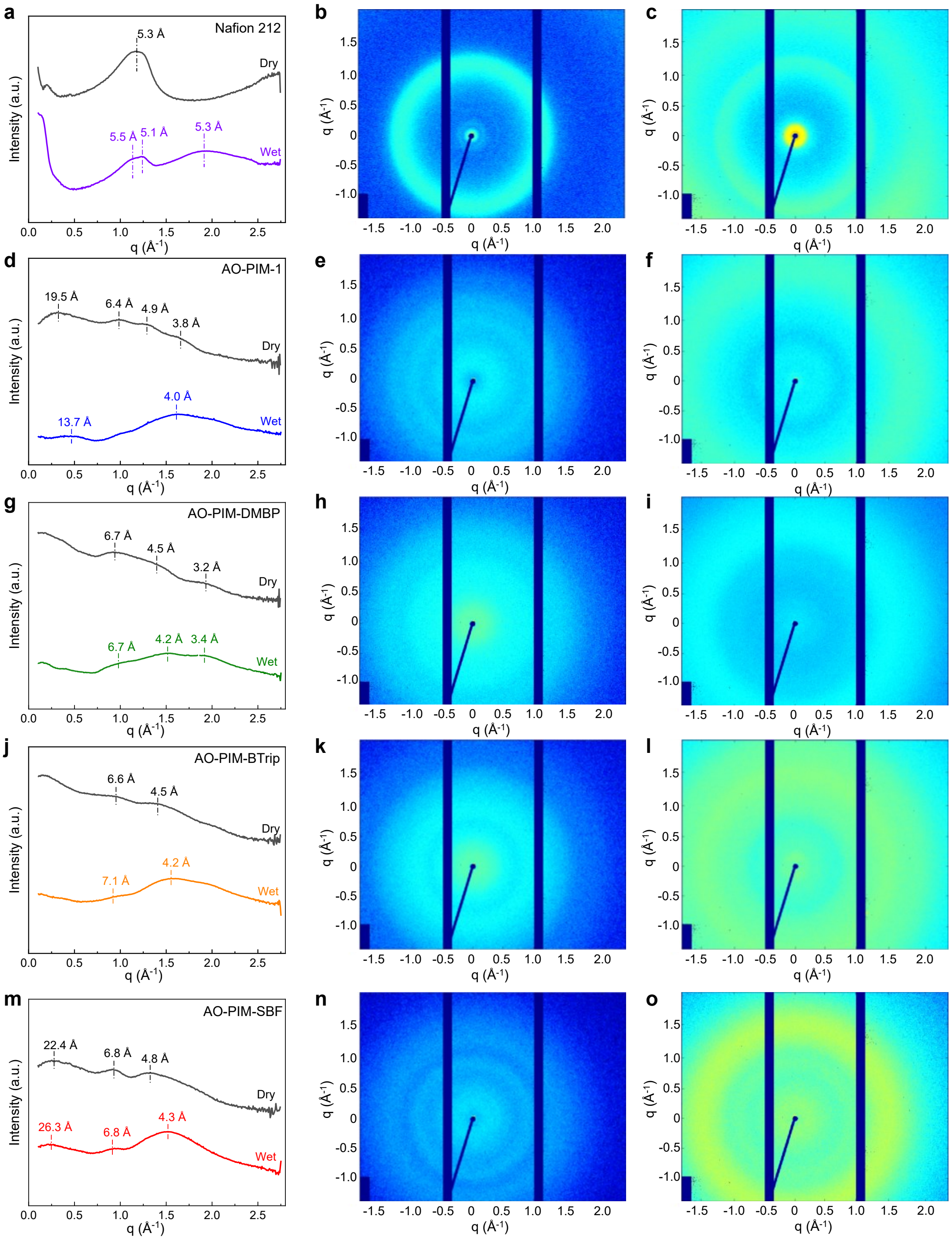

Figure S12. Wide-angle X-ray scattering (WAXS) patterns of (a-c) Nafion 212, (d-f) AO-PIM-1, (g-i) AO-PIM-DMBP, (j-l) AO-PIM-BTrip, (m-o) AO-PIM-SBF. Left: dry; Right: wet in 2D WAXS. The WAXS characterisation was performed to further investigate the micropore structure of Nafion and AO-PIM membranes in dry and wet states. Broad scattering peaks at q ~ 0.9-2.0 $Å^{-1}$ (7.2-3.2 Å in real space) were observed for dry AO-PIM membranes, which agree well with the pore information obtained from gas adsorption experiments. For wet AO-PIM membranes, the broad peaks at q ~ 0.9-2.0 $Å^{-1}$ shifted to higher q positions, indicating that the polymer network is more densely packed in wet AO-PIM membranes. This is also supported by the lack of low-q (<0.5 $Å^{-1}$) intensity. A similar phenomenon was observed and reported in our previous work[5]. In comparison, Nafion membranes presented a sharp scattering peak at q ~1.17 $Å^{-1}$ derived from the scattering among crystallites in a dry Nafion amorphous matrix.

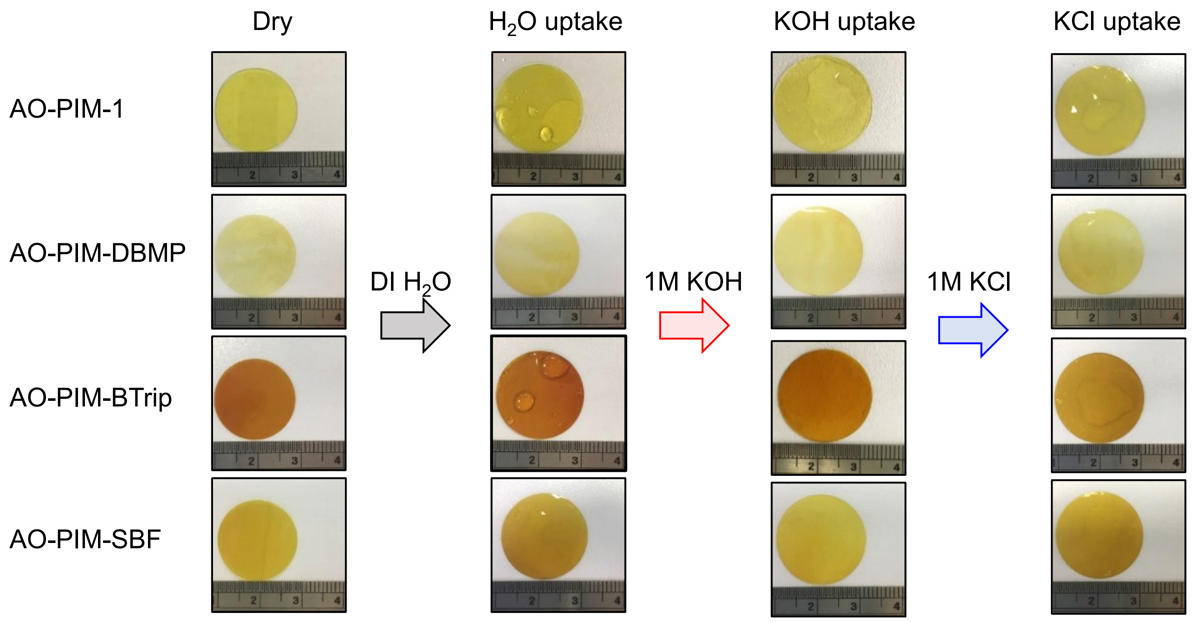

Figure S13. Photographs of AO-PIM membranes in dry and fully hydrated states. AO-PIM membranes are fully hydrated in the sequence of deionized H_2O, 1M aqueous KOH, and 1M aqueous KCl by using the same membranes.

Table S5. Summary of water/electrolytes uptake and linear swelling ratio of AO-PIM membranes.

Membranes	AO group ratio (%)	H_2O uptake (wt.%) [a]	Linear swelling ratio in H_2O (%)	KOH uptake (wt.%) [b]	Linear swelling ratio in KOH (%)	KCl uptake (wt.%) [c]	Linear swelling ratio in KCl (%)
AO-PIM-1	22.4	37.6 ±0.7	8.2 ± 0.3	100.8 ± 3.4	23.2 ± 1.5	65.3 ± 2.1	16.7 ± 1.1
AO-PIM-SBF	20.8	33.1 ±0.4	6.8 ± 0.1	60.4 ± 1.9	11.6 ± 0.8	52.4 ± 1.5	10.4 ± 0.5
AO-PIM-BTrip	20.3	32.2 ± 0.6	6.7 ± 0.2	62.6 ± 1.5	11.4 ± 0.7	51.1 ± 1.2	9.1 ± 0.7
AO-PIM-DBMP	16.9	38.5 ±0.9	9.0 ± 0.2	70.1 ± 2.3	15.9 ± 1.2	63.1 ± 1.7	16.1 ± 1.2

[a] H_2O uptake was measured by equilibrating non-deprotonated AO-PIM membranes in deionized H_2O for 24h.
[b] KOH electrolyte uptake was measured by equilibrating AO-PIM membranes (used for H_2O uptake) in 1M aqueous KOH for 24h.
[c] KCl electrolyte uptake was measured by equilibrating deprotonated AO-PIM membranes (used for KOH electrolyte uptake) in 1M aqueous KCl for 24h.

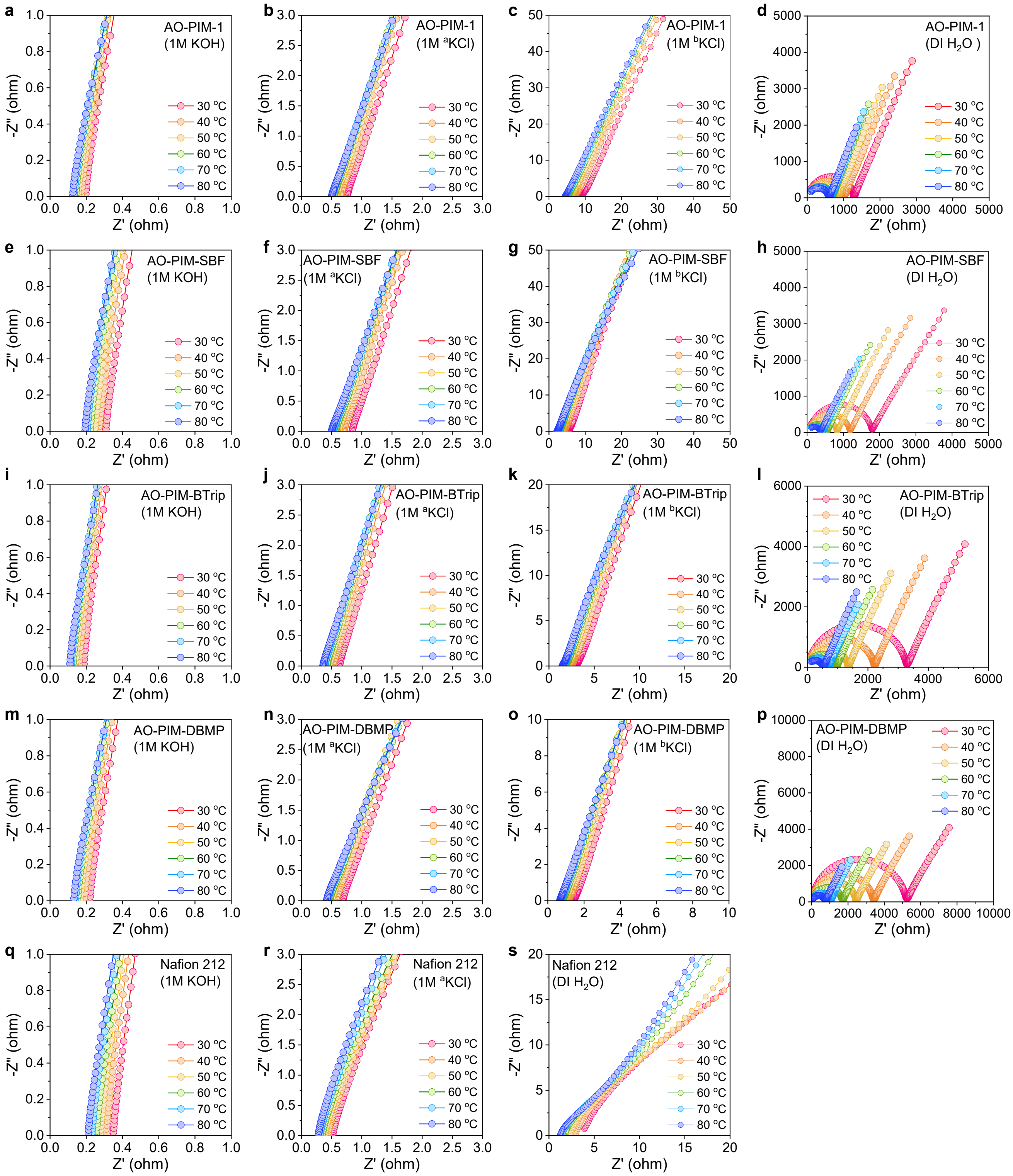

Figure S14. Nyquist plots of AO-PIM and Nafion 212 membranes measured in DI H_2O and aqueous electrolytes in the temperature range of 30-80 °C by EIS. (a)-(d) AO-PIM-1 membrane, (e)-(h) AO-PIM-SBF membrane, (i)-(l) AO-PIM-BTrip membrane, (m)-(p) AO-PIM-DBMP membrane, and (q)-(s) Nafion 212 membrane. Deprotonated PO-PIM membranes (*i.e.*, -OH form) are measured in 1M KOH, 1M ^{b}KCl and DI H_2O, while non-deprotonated membranes (*i.e.*, -O$^-$ form) in 1M ^{a}KCl.

Ionic conductivity measurements were designed to decouple two key factors, *i.e.*, free volume voids and AO groups that enable the ion transport through AO-PIM membranes. We assume that the free volume voids for ion transport would be disabled by using deionized H_2O rather KCl electrolyte, while AO groups can be activated from neutral-charged form (*i.e.*, -OH) to negative-charged form (*i.e.*, $-O^-$) by deprotonation at pH 14. *Firstly, we activated AO groups in AO-PIM membranes and measured the ionic conductivity in deionized H_2O (termed as H_2O conductivity) to study the relative contribution of AO groups for ion conduction.* All AO-PIM membranes show low H_2O conductivities in the order of 10^{-3} to 10^{-4} mS cm^{-1} with AO-PIM-1 showing the highest value and AO-PIM-DBMP showing the lowest value (Figure S15a). This trend agrees with the relative AO content of AO-PIM polymers. Lacking vehicular transport of charge carriers, the overlap of ionisable AO hydration shell is the only way to allow the charge conduction. *Similarly, we applied nonactivated AO-PIM membranes (AO groups in OH forms) in 1M aqueous KCl for ionic conductivity tests (termed as ^{b}KCl conductivity) to study the relative contribution of free volume voids for ion conduction.* The values for ^{b}KCl conductivity jump orders of magnitude to 10^{-1} to 10^0 mS cm^{-1} with AO-PIM-DBMP showing the highest value while AO-PIM-1 shows the lowest value (Figure S15b). This observed change is consistent with the relative micropore architecture that AO-PIM-DBMP exhibits the highest total pore volume hence the most sufficient pathways allowing fast transport of charge carriers. *Importantly, by activating AO groups and using KCl/KOH electrolytes together, KCl ionic conductivity (termed as ^{a}KCl conductivity) or KOH ionic conductivity (termed as KOH conductivity) of AO-PIM membranes are significantly improved to 10^1 mS cm^{-1}* that are comparable or even superior to commercial benchmark Nafion 212 membrane (Figure 2 h&i and Figure S15c).

We further normalised the H_2O conductivity and ^{b}KCl conductivity of four AO-PIM membranes to quantify the relative contribution for ion conduction from free volume voids and AO groups. AO groups contribute the largest portion to the ion conduction for AO-PIM-1 membranes, while high free volume voids contribute the largest portion to the ion conduction for AO-PIM-DBMP membranes, and AO groups and free volume voids contribute a balanced portion to the ion conduction for both AO-PIM-SBF and AO-PIM-BTrip membranes (Figure S15d). However, all of four fully activated/deprotonated AO-PIM membranes exhibit high conductivity under aqueous electrolytes, which suggests a collective property owing to the combination of high micropore volume and interaction between charge carriers and ionisable AO groups.

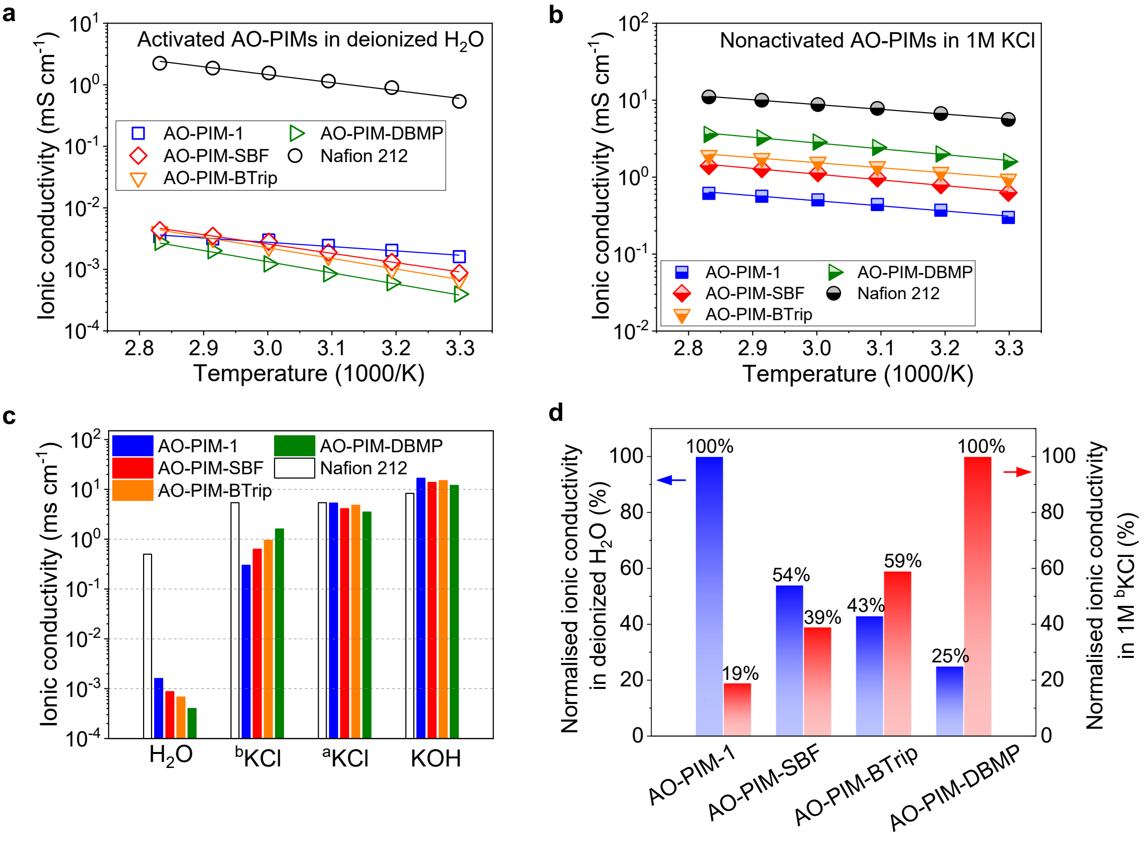

Figure S15. Ionic conductivity of AO-PIM membranes measured by EIS. Temperature dependence of ionic conductivity in the range of 30-80 °C for AO-PIM and Nafion 212 membranes measured in (a) deionized H_2O using activated membranes, and (b) 1M aqueous ^{b}KCl using nonactivated membranes. (c) Ionic conductivity of activated/nonactivated AO-PIM membranes and Nafion 212 measured in deionized H_2O, 1M KCl and 1M KOH at 30 °C. (d) Normalised ionic conductivity of activated AO-PIM membranes measured in deionized H_2O and nonactivated AO-PIM membranes measured in 1 M KCl.

Table S6. Ionic conductivity of AO-PIM membranes measured in water/aqueous electrolytes.

Membrane	KOH conductivity [a] (mS cm^{-1})	aKCl conductivity [b] (mS cm^{-1})	bKCl conductivity [c] (mS cm^{-1})	Conductivity in H_2O [d] (mS cm^{-1})
AO-PIM-1	16.7	5.3	3.0×10^{-1}	1.6×10^{-3}
AO-PIM-DBMP	12.0	3.5	1.6×10^{0}	4.0×10^{-4}
AO-PIM-SBF	13.8	4.1	6.3×10^{-1}	8.7×10^{-4}
AO-PIM-BTrip	15.0	4.8	9.5×10^{-1}	6.8×10^{-4}
Nafion 212	8.3	5.4	5.4×10^{0}	5.0×10^{-1}

[a] Deprotonated AO-PIM membranes measured in 1 M KOH aqueous solution at 30 °C.
[b] Deprotonated AO-PIM membranes measured in 1 M KCl aqueous solution at 30 °C.
[c] Non-deprotonated AO-PIM membranes measured in 1 M KCl aqueous solution at 30 °C.
[d] Deprotonated AO-PIM membranes measured in deionized H_2O at 30 °C.

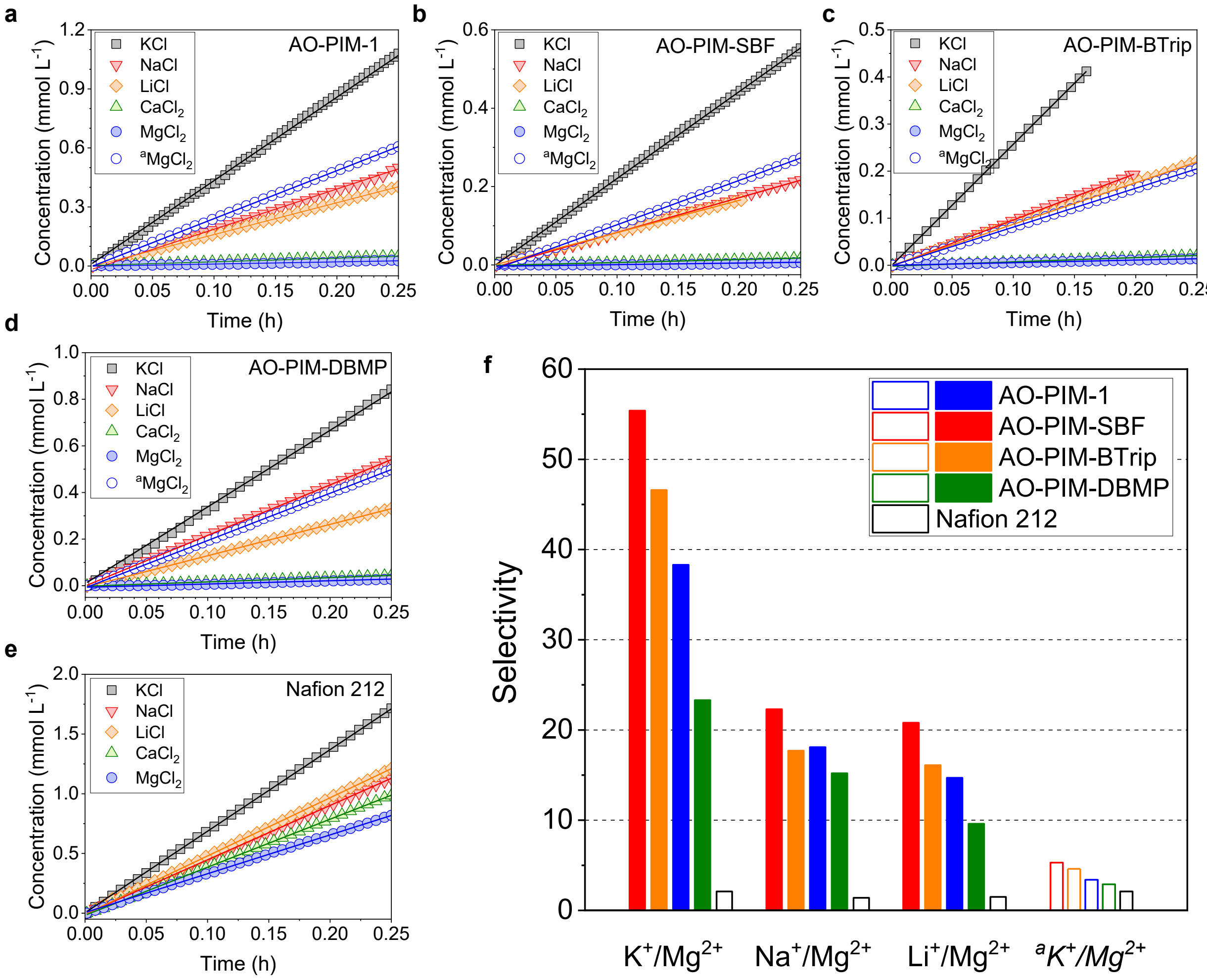

Figure S16. Ion permeation of AO-PIM and Nafion 212 membranes using concentration-driven dialysis diffusion H-cells. Concentration changes as a function of time of 1M neutral pH aqueous electrolytes in the permeate side using H-cells assembled with non-deprotonated membranes including (a) AO-PIM-1, (b) AO-PIM-SBF, (c) AO-PIM-BTrip, (d) AO-PIM-DBMP and (e) Nafion 212 membranes. (f) Ideal selectivity calculated by permeation rates through non-deprotonated AO-PIM membranes. The feed solution is 1M neutral pH aqueous electrolytes including KCl, NaCl, LiCl, $CaCl_2$, and $MgCl_2$ and the permeate side is deionized water. The $MgCl_2$ permeation rate and corresponding K^+/Mg^{2+} selectivity measured using deprotonated AO-PIMs is termed as $^aMgCl_2$ and $^a K^+/Mg^{2+}$.

Table S7. Ion permeation rates through AO-PIM and Nafion 212 membranes.

Membranes	P_{KCl} [a] (mol m^{-2} h^{-1})	P_{NaCl} [a] (mol m^{-2} h^{-1})	P_{LiCl} [a] (mol m^{-2} h^{-1})	P_{CaCl2} [a] (mol m^{-2} h^{-1})	P_{MgCl2} [a] (mol m^{-2} h^{-1})	*P_{MgCl2}* [b] *(mol m^{-2} h^{-1})*
AO-PIM-1	1.38±0.23	0.65±0.11	0.53±0.06	0.06±0.009	0.036±0.002	*0.79±0.06*
AO-PIM-DBMP	1.07±0.24	0.70±0.12	0.44±0.11	0.06±0.005	0.046±0.005	*0.66±0.05*
AO-PIM-BTrip	0.84±0.12	0.32±0.02	0.29±0.009	0.03±0.001	0.018±0.001	*0.44±0.002*
AO-PIM-SBF	0.72±0.16	0.29±0.008	0.27±0.06	0.024±0.005	0.013±0.001	*0.31±0.004*
Nafion 212	2.22±0.51	1.48±0.29	1.58±0.28	1.31±0.18	1.06±0.15	*1.06±0.05*

Table S8. Ideal selectivity of monovalent cations/magnesium ions through AO-PIM and Nafion 212 membranes.

Membranes	K^+/Mg^{2+} selectivity [a]	Na^+/Mg^{2+} selectivity[a]	Li^+/Mg^{2+} selectivity[a]	*K^+/Mg^{2+} selectivity* [b]
AO-PIM-1	38.3	18.1	14.7	*3.4*
AO-PIM-DBMP	23.3	15.2	9.6	*2.9*
AO-PIM-BTrip	46.6	17.7	16.1	*4.6*
AO-PIM-SBF	55.4	22.3	20.8	*5.3*
Nafion 212	2.1	1.4	1.5	*2.1*

[a] Permeation rates and calculated ideal selectivity are tested in 1M neutral pH aqueous electrolytes by non-deprotonated AO-PIM membranes.

[b] Permeation rates and calculated ideal selectivity are tested in 1M neutral pH aqueous electrolytes by deprotonated AO-PIM membranes (*i.e.,* pre-treated in 1M aqueous KOH overnight).

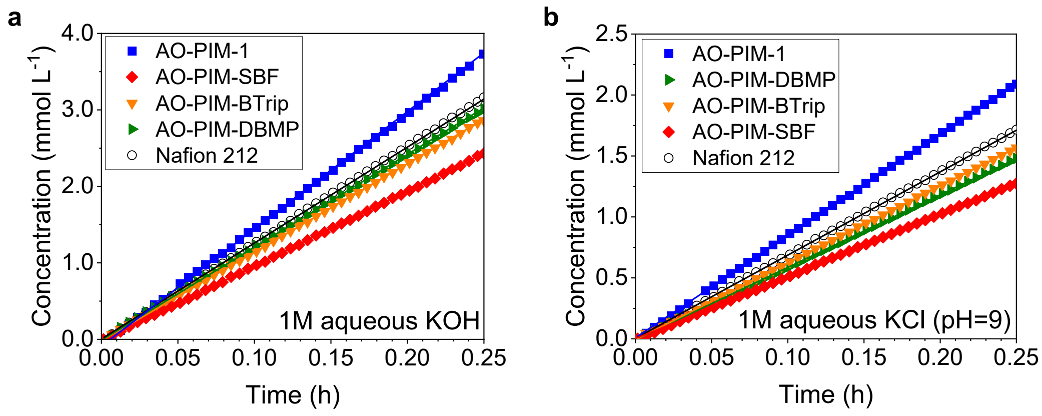

Figure S17. Permeation of small charge-carrying ions through AO-PIM membranes measured by dialysis diffusion H-cells. Concentration changes as a function of time of (a) 1M aqueous KOH and (b) 1M aqueous KCl (pH = 9) in the permeate side using dialysis diffusion H-cells assembled with deprotonated AO-PIM membranes. The permeate side is deionized H_2O.

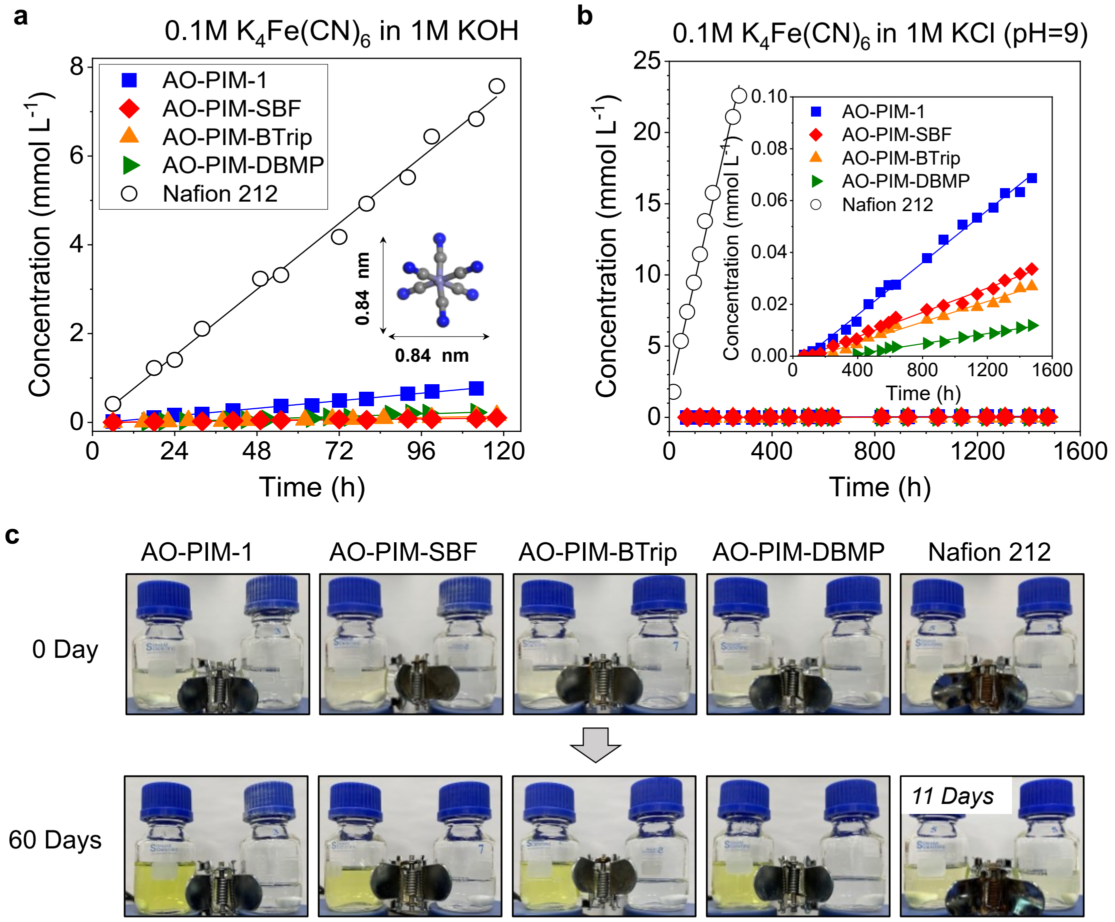

Figure S18. $K_4Fe(CN)_6$ crossover through AO-PIM membranes measured by dialysis diffusion H-cells. Concentration changes as a function of time of (a) 0.1M $K_4Fe(CN)_6$ in 1M aqueous KOH and (b) 0.1M $K_4Fe(CN)_6$ in 1M aqueous KCl in the permeate side using dialysis diffusion H-cells assembled with deprotonated AO-PIM membranes. The electrolyte in both feed and permeate side is 1M aqueous KOH with pH = 14 for (a) and 1M KCl with pH = 9 for (b). (c) Photographs showing the colour change of permeate solution at the beginning (0 day) and the end (60 days for AO-PIM and 11 days for Nafion 212 membranes) in (b). Colourless KCl solution maintained in the permeate side indicates the slow $K_4Fe(CN)_6$ crossover rate through AO-PIM membranes during 60-day period, however, $K_4Fe(CN)_6$ crossover rate is at least one order of magnitude faster through Nafion 212 membrane based on the obvious colour change observed in permeate side of the otherwise identical Nafion 212 H cell even in 11 days.

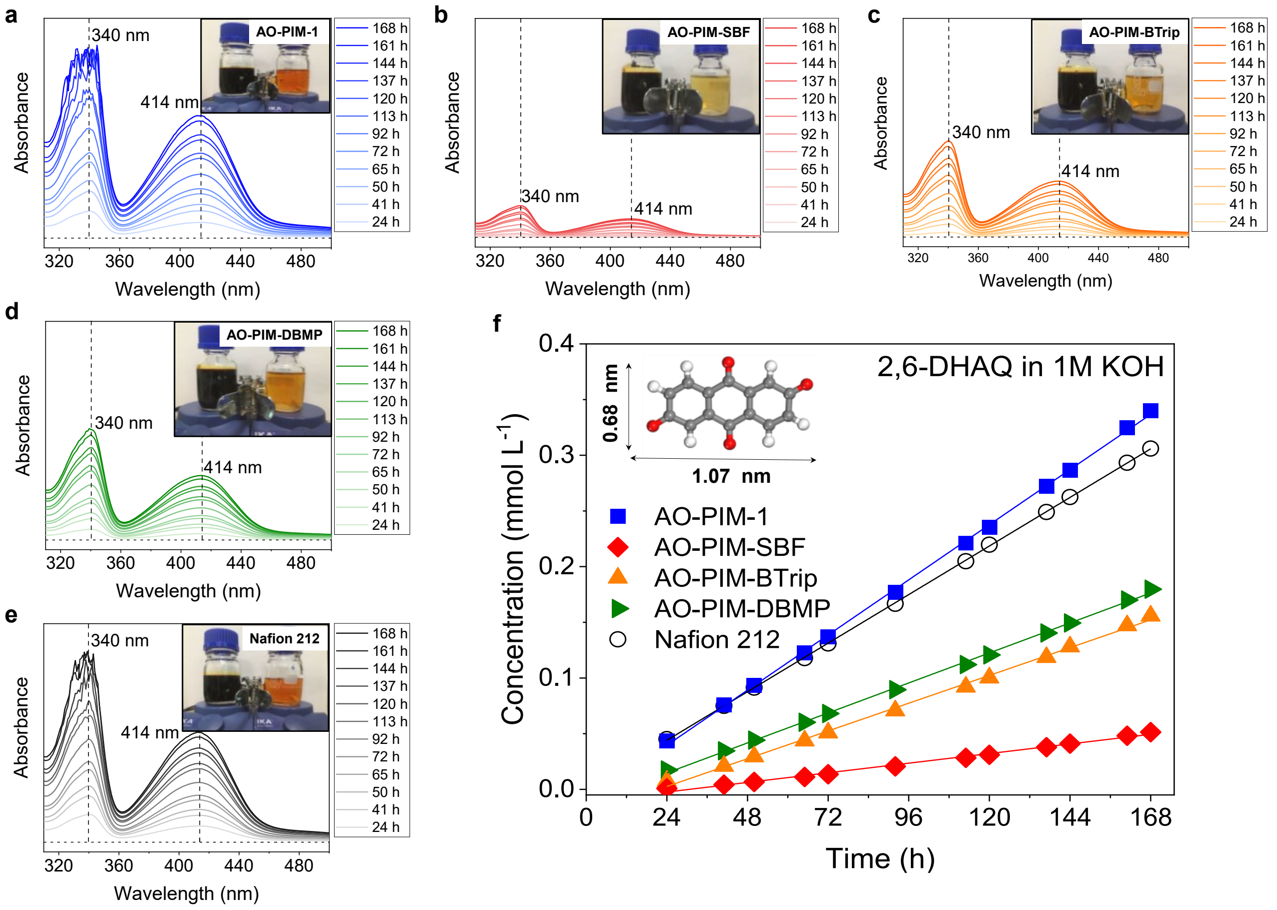

Figure S19. 2,6-DHAQ crossover through AO-PIM membranes using dialysis diffusion H-cells. UV-Vis spectra of 2,6-DHAQ as a function of time in the permeate side using H-cells assembled with (a) AO-PIM-1 (b) AO-PIM-SBF (c) AO-PIM-BTrip, (d) AO-PIM-DBMP and (e) Nafion 212 membranes. Inset photographs show the colours of feed and permeate solution after 168-hour testing period. (f) Concentration changes as a function of time of 0.1M 2,6-DHAQ in the permeate side using dialysis diffusion H-cells assembled with deprotonated AO-PIM membranes. The electrolyte is 1M aqueous KOH at feed and permeate side.

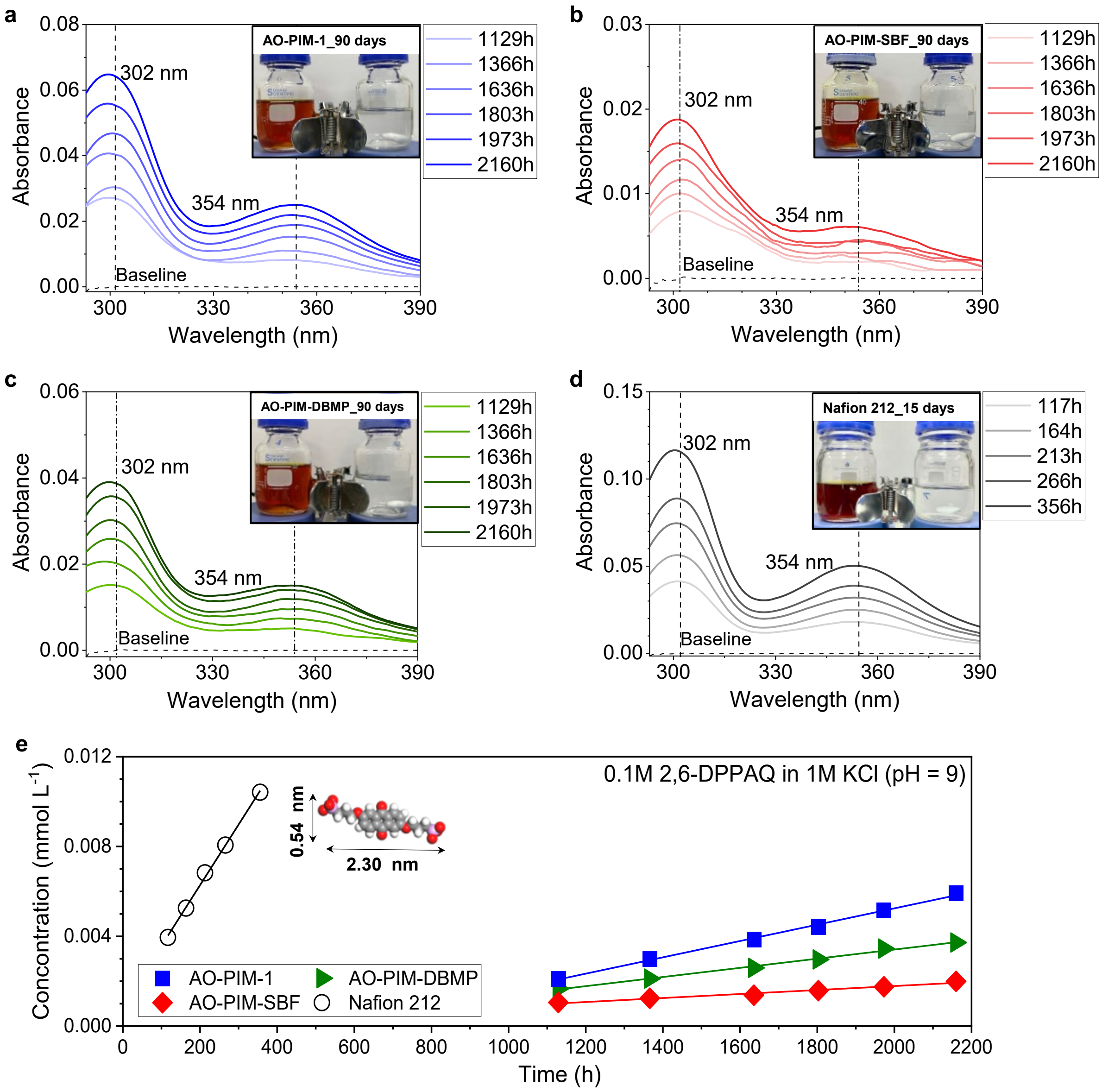

Figure S20. 2,6-DPPAQ crossover through AO-PIM membranes using dialysis diffusion H-cells. UV-Vis spectra of 2,6-DPPAQ as a function of time in the permeate side using H-cells assembled with (a) AO-PIM-1 (b) AO-PIM-SBF (c) AO-PIM-DBMP and (d) Nafion 212 membranes. Inset photographs show the colours of feed and permeate solution after 90-day testing period for AO-PIM and 15-day period for Nafion 212 membranes). (e) Concentration changes as a function of time of 0.1M 2,6-DPPAQ in the permeate side using dialysis diffusion H-cells assembled with deprotonated AO-PIM membranes. The electrolyte is 1M aqueous KCl (pH = 9) at feed and permeate side.

Table S9. KOH permeation, and $K_4Fe(CN)_6$, 2,6-DHAQ crossover through deprotonated AO-PIM membranes.

Membranes	KOH		$K_4Fe(CN)_6$		2,6-DHAQ	
	Permeation rate (mol m^{-2} h^{-1})	Diffusivity (cm^2 s^{-1})	Permeation rate (mol m^{-2} h^{-1})	Diffusivity (cm^2 s^{-1})	Permeation rate (mol m^{-2} h^{-1})	Diffusivity (cm^2 s^{-1})
Nafion 212	4.07±0.35	6.3×10^{-6}	2.0×10^{-2}	2.8×10^{-8}	7.7×10^{-4}	1.2×10^{-9}
AO-PIM-1	4.98±1.07	6.6×10^{-6}	2.3×10^{-3}	3.1×10^{-9}	8.7×10^{-4}	1.7×10^{-9}
AO-PIM-DBMP	3.95±0.52	4.8×10^{-6}	7.4×10^{-4}	1.3×10^{-9}	3.6×10^{-4}	9.5×10^{-10}
AO-PIM-BTrip	3.76±0.21	4.4×10^{-6}	4.1×10^{-4}	6.2×10^{-10}	3.4×10^{-4}	4.5×10^{-10}
AO-PIM-SBF	3.19±0.38	3.6×10^{-6}	2.7×10^{-4}	3.5×10^{-10}	1.2×10^{-4}	1.6×10^{-10}

Table S10. KCl permeation, and $K_4Fe(CN)_6$, 2,6-DPPAQ crossover through deprotonated AO-PIM membranes.

Membranes	KCl		$K_4Fe(CN)_6$		2,6-DPPAQ	
	Permeation rate (mol m^{-2} h^{-1})	Diffusivity (cm^2 s^{-1})	Permeation rate (mol m^{-2} h^{-1})	Diffusivity (cm^2 s^{-1})	Permeation rate (mol m^{-2} h^{-1})	Diffusivity (cm^2 s^{-1})
Nafion 212	2.22±0.51	3.4×10^{-6}	1.6×10^{-2}	2.2×10^{-8}	7.0×10^{-6}	9.8×10^{-12}
AO-PIM-1	2.71±0.21	3.6×10^{-6}	9.8×10^{-6}	1.2×10^{-11}	9.4×10^{-7}	7.9×10^{-13}
AO-PIM-DBMP	1.93±0.36	2.4×10^{-6}	2.0×10^{-6}	4.8×10^{-12}	5.3×10^{-7}	4.7×10^{-13}
AO-PIM-BTrip	2.05±0.14	2.4×10^{-6}	3.9×10^{-6}	4.3×10^{-12}	-	-
AO-PIM-SBF	1.65±0.08	1.9×10^{-6}	4.6×10^{-6}	4.3×10^{-12}	1.7×10^{-7}	1.4×10^{-13}

Table S11. Selectivity of charge-carrying ions and redox species through deprotonated AO-PIM membranes.

Membranes	Selectivity (KOH/$K_4Fe(CN)_6$)	Selectivity (KOH/2,6-DHAQ)	Selectivity (KCl/$K_4Fe(CN)_6$)	Selectivity (KCl/2,6-DPPAQ)
Nafion 212	2.0×10^{2}	5.3×10^{3}	1.4×10^{2}	3.2×10^{5}
AO-PIM-1	2.2×10^{3}	5.7×10^{3}	2.8×10^{5}	2.9×10^{6}
AO-PIM-DBMP	5.3×10^{3}	1.1×10^{4}	9.5×10^{5}	3.6×10^{6}
AO-PIM-BTrip	9.2×10^{3}	1.1×10^{4}	5.3×10^{5}	-
AO-PIM-SBF	1.2×10^{4}	2.7×10^{4}	3.6×10^{5}	9.5×10^{6}

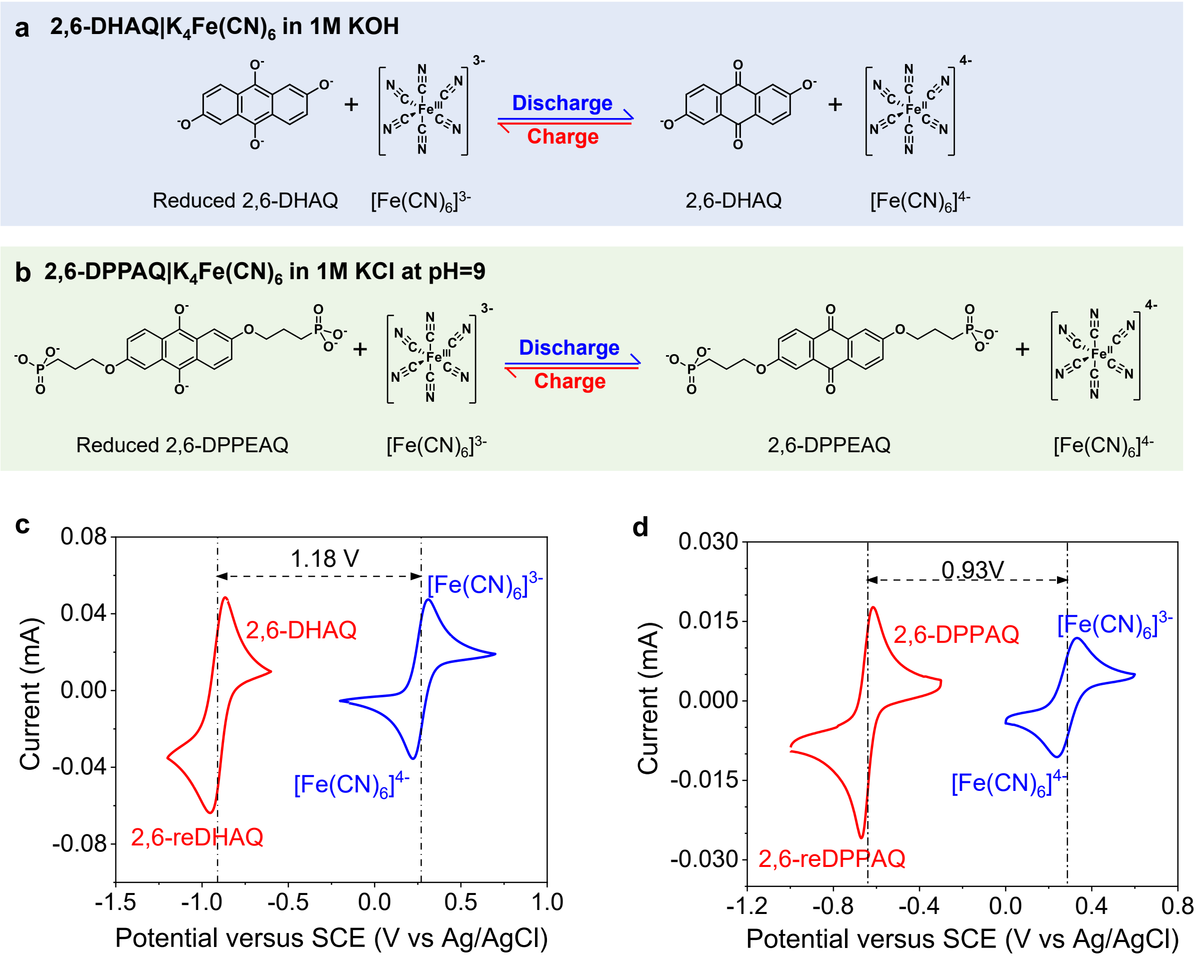

Figure S21. Electrochemical reaction mechanism of (a) 2,6-DHAQ|$K_4Fe(CN)_6$ redox couple in 1 M aqueous KOH and (b) 2,6-DPPAQ|$K_4Fe(CN)_6$ redox couple in 1M aqueous KCl at pH = 9. Typical C-V curves of (c) 2,6-DHAQ (red trace) and $K_4Fe(CN)_6$ (blue trace) in 1M aqueous KOH[5] and (d) 2,6-DPPAQ (red trace) and $K_4Fe(CN)_6$ (blue trace) in 1M aqueous KCl at pH = 9 using three-electrode electrochemical systems.

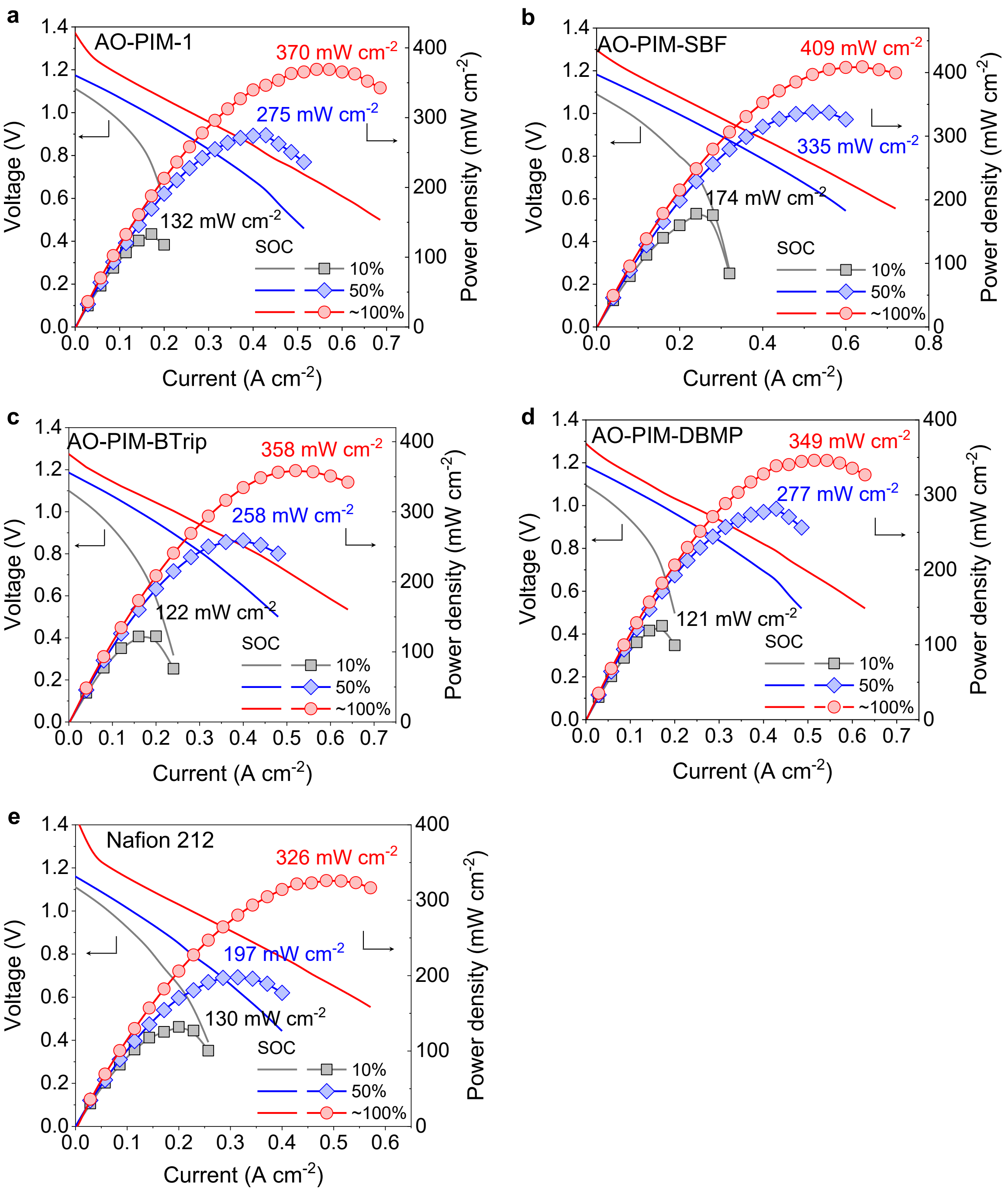

Figure S22. Polarization curves at 20, 50, and ~100% SOC measured in RFB full cells using 2,6-DHAQ (0.1M) | $K_4Fe(CN)_6$ (0.1M) configured with AO-PIM and Nafion 212 membranes.

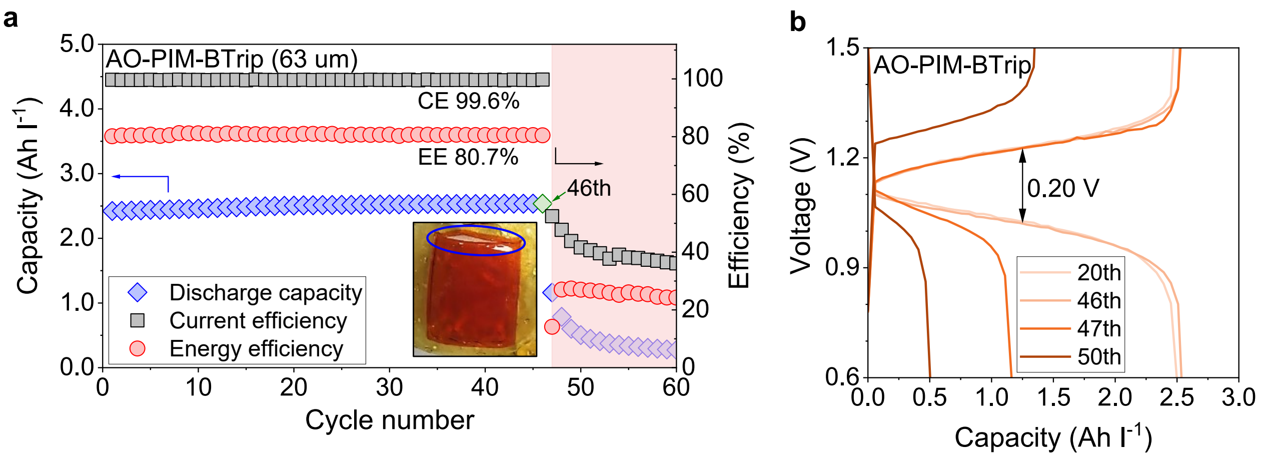

Figure S23. (a) Cycling performance and (b) charging-discharging curves of 2,6-DHAQ (0.1M) | $K_4Fe(CN)_6$ (0.1M) RFB full cell configured with AO-PIM-BTrip membrane. AO-PIM-BTrip cell retains 95% of theoretical discharge capacity in the initial 46 cycles whilst dropping to only ~10% capacity retention at the 60th cycle. Inset photograph shows obvious DHAQ adsorption in the whole active area and crack at the edge of membrane active area which leads to a dramatic capacity decay.

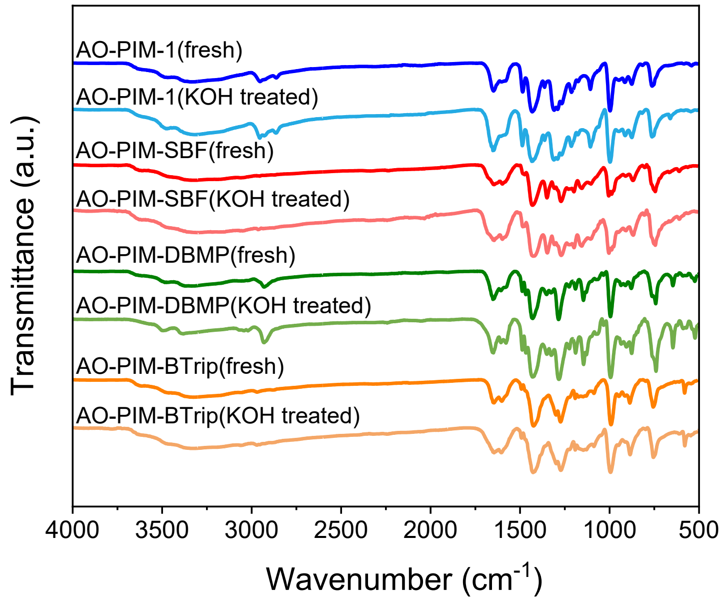

Figure S24. FTIR spectra of fresh AO-PIM polymers and alkaline-treated AO-PIM polymers in 3M aqueous KOH at R.T for 4 weeks.

Table S12. N_2 adsorption and GPC results of fresh and KOH-treated AO-PIM polymers.

Polymers	SA_{BET} [a] (m^2 g^{-1})	SA_{BET} [b] (m^2 g^{-1})	Mw [a] (kDa)	Mw[b] (kDa)	PDI [a]	PDI [b]
AO-PIM-1	567 [c]	548	205.2	189.6	1.74	2.01
AO-PIM-SBF	611	665	170.2	161.3	2.08	2.32
AO-PIM-BTrip	649	595	145.7	162.4	1.94	1.87
AO-PIM-DBMP	608	636	99.1	87.1	2.04	2.31

[a] SA_{BET}, Mw and PDI values were obtained from fresh AO-PIM membranes.
[b] SA_{BET}, Mw and PDI values were obtained from AO-PIM membranes after 3M aqueous KOH treatment at R.T for 4 weeks.
[c] SA_{BET} value of AO-PIM-1 was from our previously reported work[5].

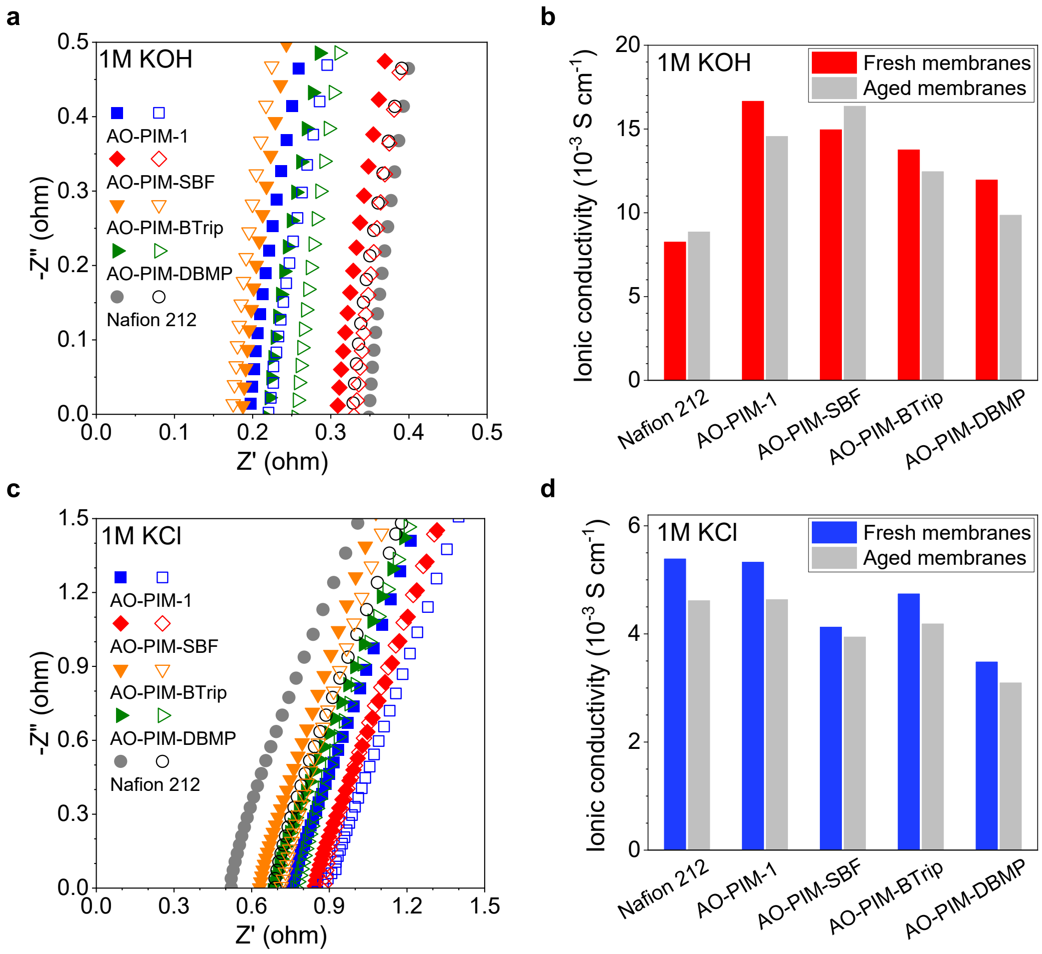

Figure S25. Nyquist plots and ionic conductivity values of fresh and ~180-day-aged AO-PIM and Nafion 115 membranes measured at 30 °C in (a) (b) 1M aqueous KOH and (c) (d) 1M aqueous KCl (pH = 9). Similar EIS spectra of each membrane in fresh and aged stage suggests the retention of microporous structure of swollen hydrophilic AO-PIM membranes. This is because typical relaxation of glassy polymers including PIMs from nonequilibrium to equilibrium packing state over time generally leads to significant reduction of free volume (*i.e.* aging behaviour) hence the deterioration of electrochemical performance including ionic conductivity[11].

Table S13. Area specific resistance (ASR) and ionic conductivity of fresh and ~180-day-aged AO-PIM and Nafion membranes..

Membranes	ASR in KOH (Ω cm^2) [a]		KOH conductivity (10^{-3} S cm^{-1}) [b]		ASR in KCl (Ω cm^2) [c]		KCl Conductivity (10^{-3} S cm^{-1}) [b]	
	Fresh	Aged	Fresh	Aged	Fresh	Aged	Fresh	Aged
AO-PIM-1	0.39	0.43	16.7	14.6	1.48	1.69	5.3	4.6
AO-PIM-SBF	0.37	0.34	15.0	16.4	1.24	1.39	4.8	4.2
AO-PIM-BTrip	0.60	0.65	13.8	12.5	1.65	1.72	4.1	4.0
AO-PIM-DBMP	0.43	0.50	12.0	9.9	1.34	1.50	3.5	3.1
Nafion 212	0.68	0.64	8.3	8.9	1.01	1.16	5.4	4.6

[a] ASR was measured by EIS in 1 M aqueous KOH at 30 °C with membrane active area of 2.00 cm^2.
[b] Ionic conductivity of AO-PIM and Nafion membranes derived from its corresponding EIS spectra.
[c] ASR was measured by EIS in 1 M aqueous KCl at 30 °C with membrane active area of 2.00 cm^2.

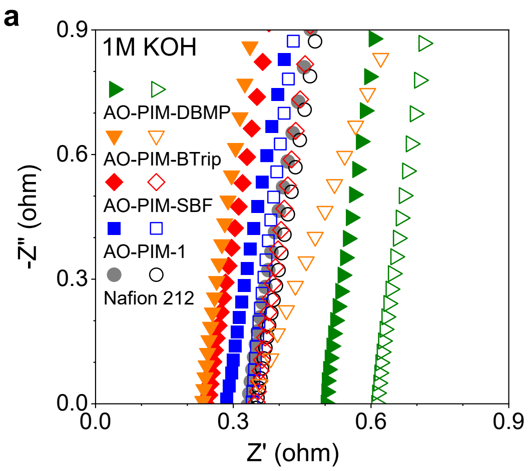

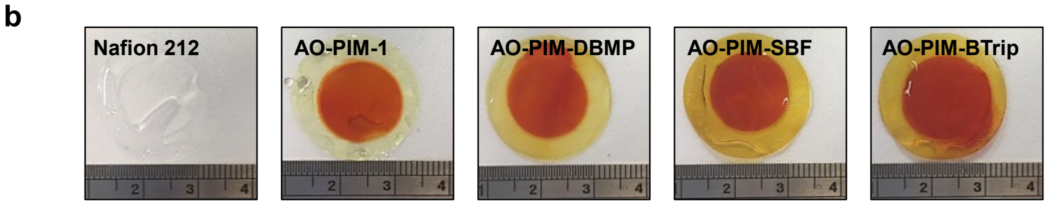

Figure S26. (a) Nyquist plots of fresh and DHAQ-adsorbed AO-PIM and Nafion 115 membranes measured at 30 °C. (b) Photographs of AO-PIM and Nafion 115 membranes after 0.1M 2,6-DHAQ adsorption in 1M aqueous KOH for 7 days.

Table S14. ASR and ionic conductivity of fresh and DHAQ-adsorbed AO-PIM and Nafion membranes.

Membranes	ASR in 1M KOH (Ω cm^2) [a]		KOH Conductivity (10^{-3} S cm^{-1}) [b]	
	Fresh	DHAQ-adsorbed [c]	Fresh	DHAQ-adsorbed [c]
Nafion 212	0.65	0.69	8.8	8.2
AO-PIM-1	0.56	0.67	10.5	8.5
AO-PIM-SBF	0.48	0.69	12.5	8.2
AO-PIM-BTrip	0.46	0.67	13.3	8.5
AO-PIM-DBMP	0.98	1.20	5.5	4.5

[a] ASR)was measured by EIS in 1 M aqueous KOH at 30 °C with membrane active area of 2.00 cm^2.
[b] Ionic conductivity values of membranes derived from EIS spectra.
[c] Membranes were soaked in 0.1M 2,6-DHAQ for 7 days before ASR measurements. (*i.e.,* membranes after 2,6-DHAQ diffusion dialysis tests were used as DHAQ-absorbed membranes for ASR measurements).

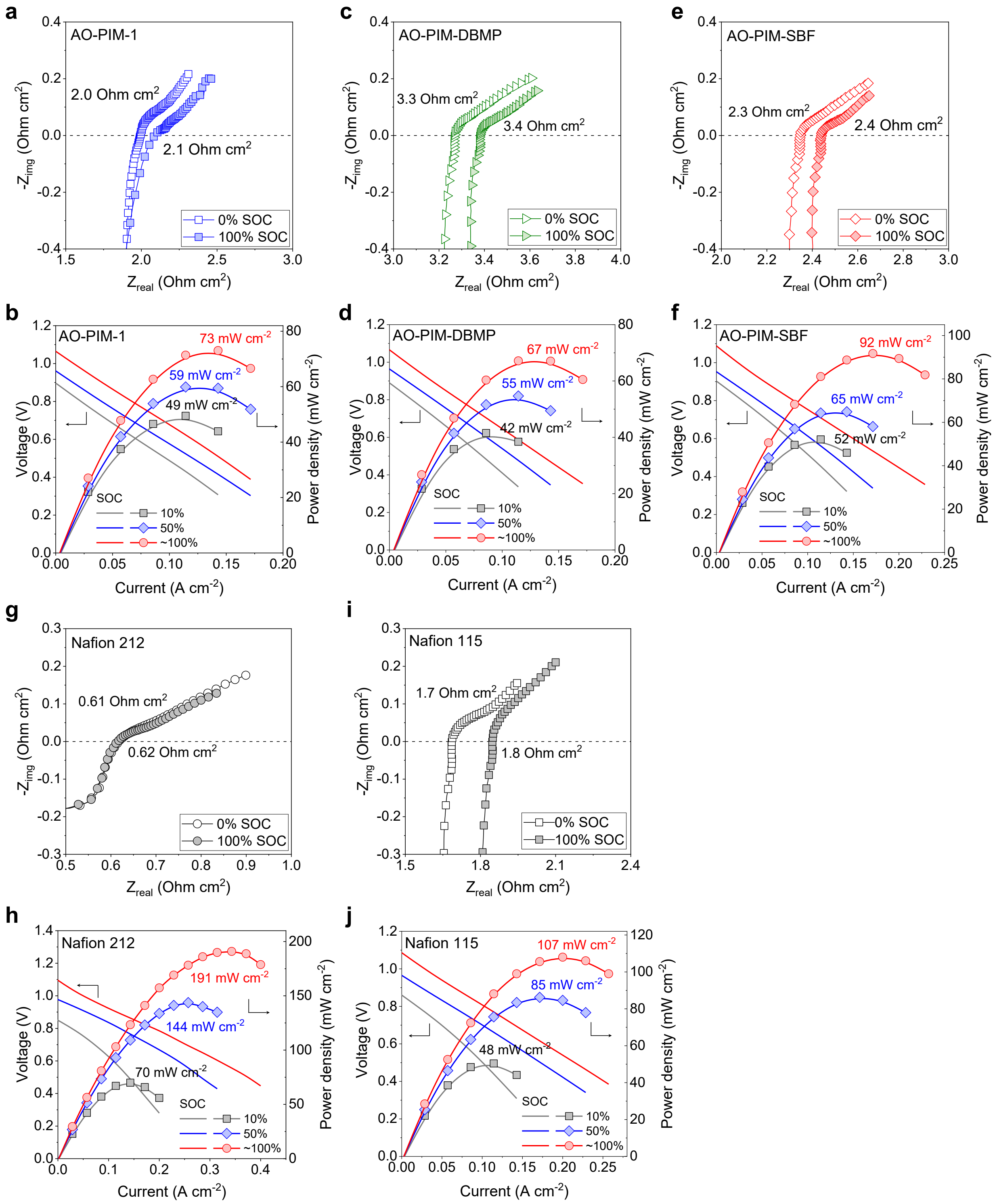

Figure S27. EIS spectra at 0 and 100% SOC and polarization curves at 20, 50, and ~100% SOC measured in RFB full-cells using $K_4Fe(CN)_6$ (0.1 M)|2,6-DPPAQ (0.1 M) configured with (a) (b) AO-PIM-1 (c) (d) AO-PIM-DBMP (e) (f) AO-PIM-SBF, (g) (h) Nafion 212, and (i) (j) Nafion 115 membranes.

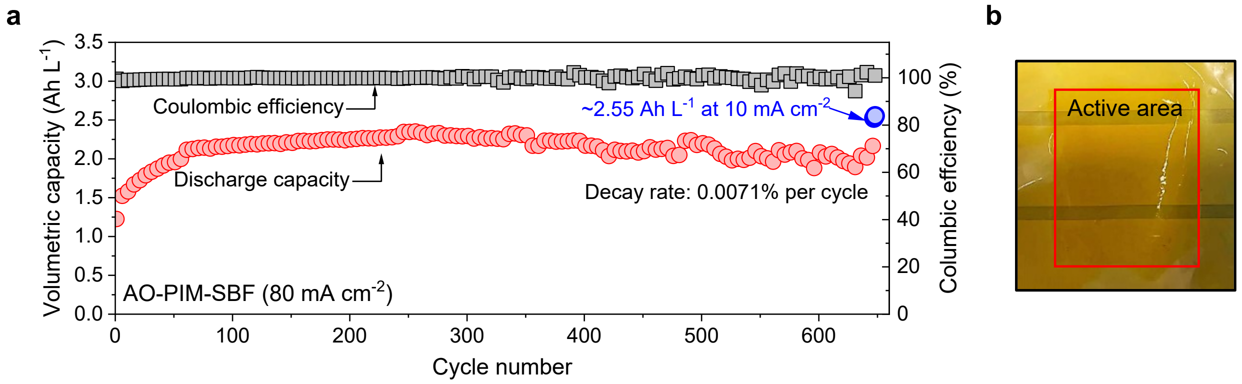

Figure S28. Volumetric capacity and coulombic efficiency of $K_4Fe(CN)_6$ (0.1M)|2,6-DPPAQ (0.1M) RFB full-cell assembled with AO-PIM-SBF membrane in 1M aqueous KCl at pH 9 and a current density of 80 mA cm^{-2}. **(b)** A photograph showing the adsoprtion condition of AO-PIM-SBF membrane after a long-term cycling test. AO-PIM-SBF cell maintains 95.1% of its theoretical capacity after 650 cycles at 80 mA cm^{-2} with coulombic efficiency steady over 99%, showing the capacity decay rate as low as 0.0071% per cycle or the average capacity retention rate up to 99.9929% per cycle, which is much more stable to the otherwise identical AO-PIM-SBF cell using the 2,6-DHAQ|$K_4Fe(CN)_6$ redox couple in 1M aqueous KOH.

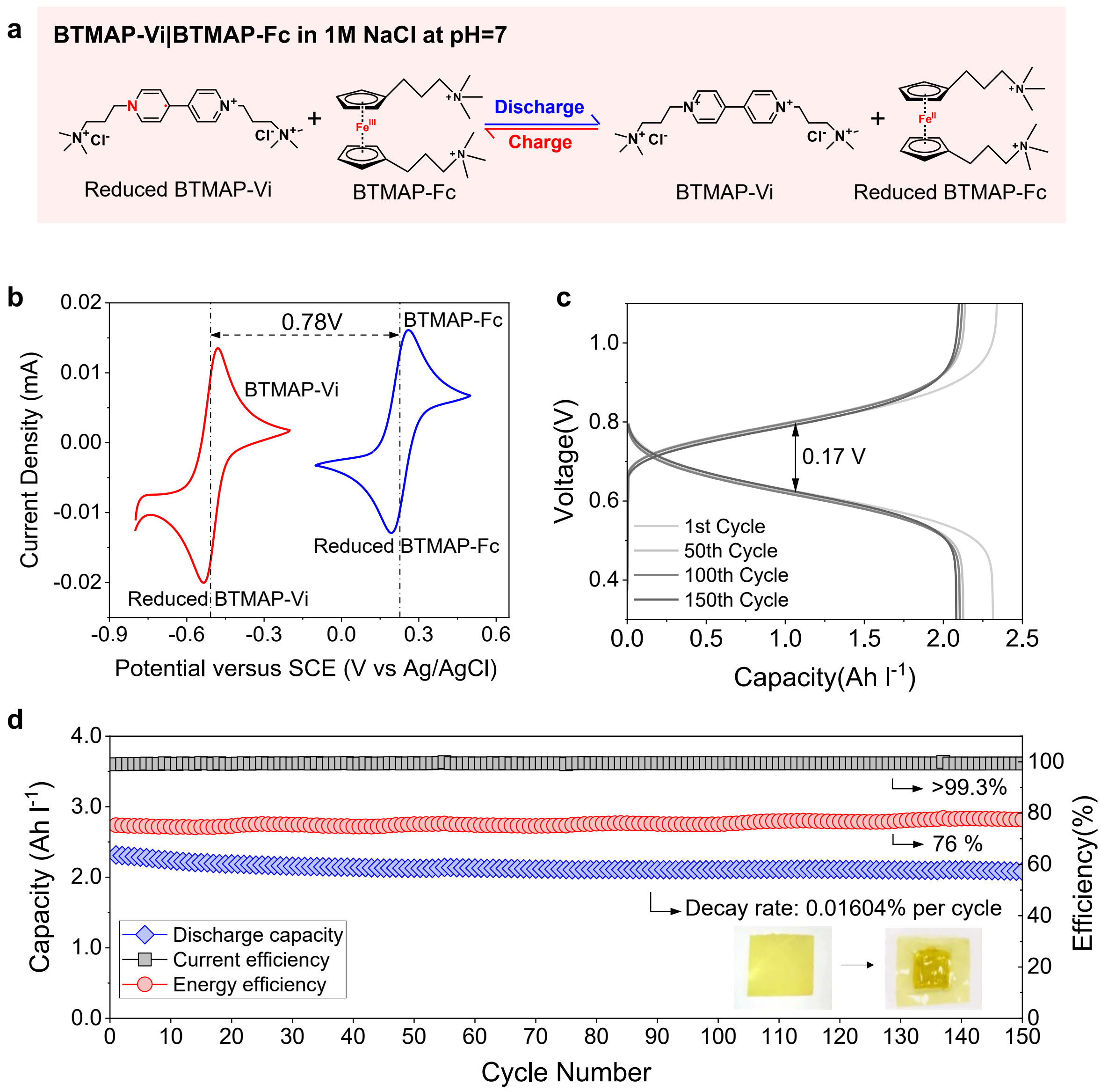

Figure S29. The BTMAP-Vi|BTMAP-Fc RFB full-cell using AO-PIM-1 membrane. (a) Electrochemical reaction mechanism of BTMAP-Vi|BTMAP-Fc redox couple in 1M aqueous NaCl at pH=7. (b) Typical C-V curves of BTMAP-Vi (red trace) and BTMAP-Vi (blue trace) in 1M aqueous KCl at pH=7 using three-electrode electrochemical systems. (c) Representative charging-discharging curves of A BTMAP-Vi (0.1M) | BTMAP-Fc (0.1M) RFB full-cell configured with AO-PIM-1 membrane. (d) Volumetric capacity, coulombic efficiency and energy efficiency of a BTMAP-Vi (0.1M) | BTMAP-Fc (0.1M) RFB full-cell assembled with AO-PIM-1 membranes in 1M aqueous KCl at pH 7 and a current density of 25 mA cm^{-2}. The AO-PIM-1 cell assembled with a positively-charged but bulky BTMAP-Vi|BTMAP-Fc redox couple achieved a high coulombic efficiency of >99.3% and energy efficiency of 76.0%, but also showing high cycling stability with electrochemical capacity retentions of 90.0% over 150 cycles at 25 mA cm^{-2}. The positive charge of this bis(trimethylammoniumpropyl) functionalities and negative charge of activated AO groups (Figure S2 and Table S1) might have been expected to encourage severe absorption of this redox couple into the AO-PIM-1 membrane. However, the relatively stable battery performance suggests that the strong size-sieving effect of AO-PIM membranes plays a key role in achieving high molecular selectivity.

`

References

[1] P. M. Budd, E. S. Elabas, B. S. Ghanem, S. Makhseed, N. B. McKeown, K. J. Msayib, C. E. Tattershall, D. Wang, *Adv. Mater.* **2004**, *16*, 456-459.
[2] C. G. Bezzu, M. Carta, A. Tonkins, J. C. Jansen, P. Bernardo, F. Bazzarelli, N. B. McKeown, *Adv. Mater.* **2012**, *24*, 5930-5933.
[3] B. Comesaña-Gándara, J. Chen, C. G. Bezzu, M. Carta, I. Rose, M.-C. Ferrari, E. Esposito, A. Fuoco, J. C. Jansen, N. B. McKeown, *Energy Environ. Sci.* **2019**, *12*, 2733-2740.
[4] H. A. Patel, C. T. Yavuz, *Chem. Commun.* **2012**, *48*, 9989-9991.
[5] R. Tan, A. Wang, R. Malpass-Evans, R. Williams, E. W. Zhao, T. Liu, C. Ye, X. Zhou, B. P. Darwich, Z. Fan, L. Turcani, E. Jackson, L. Chen, S. Y. Chong, T. Li, K. E. Jelfs, A. I. Cooper, N. P. Brandon, C. P. Grey, N. B. McKeown, Q. Song, *Nat. Mater.* **2020**, *19*, 195-202.
[6] M. J. Frisch, G. W. Trucks, H. B. Schlegel, G. E. Scuseria, M. A. Robb, J. R. Cheeseman, G. Scalmani, V. Barone, G. A. Petersson, H. Nakatsuji, X. Li, M. Caricato, A. V. Marenich, J. Bloino, B. G. Janesko, R. Gomperts, B. Mennucci, H. P. Hratchian, J. V. Ortiz, A. F. Izmaylov, J. L. Sonnenberg, Williams, F. Ding, F. Lipparini, F. Egidi, J. Goings, B. Peng, A. Petrone, T. Henderson, D. Ranasinghe, V. G. Zakrzewski, J. Gao, N. Rega, G. Zheng, W. Liang, M. Hada, M. Ehara, K. Toyota, R. Fukuda, J. Hasegawa, M. Ishida, T. Nakajima, Y. Honda, O. Kitao, H. Nakai, T. Vreven, K. Throssell, J. A. Montgomery Jr., J. E. Peralta, F. Ogliaro, M. J. Bearpark, J. J. Heyd, E. N. Brothers, K. N. Kudin, V. N. Staroverov, T. A. Keith, R. Kobayashi, J. Normand, K. Raghavachari, A. P. Rendell, J. C. Burant, S. S. Iyengar, J. Tomasi, M. Cossi, J. M. Millam, M. Klene, C. Adamo, R. Cammi, J. W. Ochterski, R. L. Martin, K. Morokuma, O. Farkas, J. B. Foresman, D. J. Fox, Wallingford, CT, **2016**.
[7] a) A. D. Becke, *Phys. Rev. A* **1988**, *38*, 3098-3100; b) C. Lee, W. Yang, R. G. Parr, *Physical Review B* **1988**, *37*, 785-789.
[8] W. R. Wadt, P. J. Hay, *The Journal of Chemical Physics* **1985**, *82*, 284-298.
[9] A. Alesadi, W. Xia, *Macromolecules* **2020**, *53*, 2754-2763.
[10] a) A. Kusoglu, A. Z. Weber, *Chem. Rev.* **2017**, *117*, 987-1104; b) S. P. Fernandez Bordín, H. E. Andrada, A. C. Carreras, G. E. Castellano, R. G. Oliveira, V. M. Galván Josa, *Polymer* **2018**, *155*, 58-63.
[11] C. Li, S. M. Meckler, Z. P. Smith, J. E. Bachman, L. Maserati, J. R. Long, B. A. Helms, *Adv. Mater.* **2018**, *30*, 1704953-1704985.

Author Contributions

Q.S. and N.B.M. conceived and supervised the project. C.Y. synthesized and characterized membranes. A.W. carried out characterizations of membranes. R.T. performed RFB tests. J.C., B.C.G., and C.G.B helped with polymer synthesis. A. A-F, and S. G. performed SAXS/WAXS measurements and data analysis, Z. F. and J.W. helped with experiments. C.B., and K.E.J. performed modelling. N.P.B and A.R.K participated in the discussion of results. Q.S., N.B.M., and C.Y. wrote the manuscript with contributions from all authors. All authors discussed the results and commented on the manuscript at all stages.
